# Impact of *pe_pgrs33* Gene Polymorphisms on *Mycobacterium tuberculosis* Infection and Pathogenesis

**DOI:** 10.3389/fcimb.2017.00137

**Published:** 2017-04-21

**Authors:** Serena Camassa, Ivana Palucci, Raffaella Iantomasi, Tiziana Cubeddu, Mariachiara Minerva, Flavio De Maio, Samuel Jouny, Elisa Petruccioli, Delia Goletti, Francesco Ria, Michela Sali, Maurizio Sanguinetti, Riccardo Manganelli, Stefano Rocca, Priscille Brodin, Giovanni Delogu

**Affiliations:** ^1^Institute of Microbiology, Università Cattolica del Sacro Cuore - Fondazione Policlinico Universitario GemelliRome, Italy; ^2^Univ. Lille, Centre National de la Recherche Scientifique, Institut National de la Santé et de la Recherche Médicale, CHU Lille, Institut Pasteur de Lille, U1019 - UMR 8204 - CIIL - Centre d'Infection et d'Immunité de LilleLille, France; ^3^Department of Veterinary Medicine, University of SassariSassari, Italy; ^4^Translational Research Unit, Department of Epidemiology and Preclinical Research, “Lazzaro Spallanzani” National Institute for Infectious DiseasesRome, Italy; ^5^Institute of General Pathology, Università Cattolica del Sacro Cuore - Fondazione Policlinico Universitario GemelliRome, Italy; ^6^Department of Molecular Medicine, University of PaduaPadua, Italy

**Keywords:** *Mycobacterium tuberculosis*, PE_PGRS, genetic variability, polymorphisms, bacterial pathogenesis, host-pathogen interactions

## Abstract

PE_PGRS33 is a surface-exposed protein of *Mycobacterium tuberculosis* (*Mtb*) which exerts its role in macrophages entry and immunomodulation. In this study, we aimed to investigate the polymorphisms in the *pe_pgrs33* gene of *Mtb* clinical isolates and evaluate their impact on protein functions. We sequenced *pe_pgrs33* in a collection of 135 clinical strains, genotyped by 15-loci MIRU-VNTR and spoligotyping and belonging to the *Mtb* complex (MTBC). Overall, an association between *pe_pgrs33* alleles and MTBC genotypes was observed and a dN/dS ratio of 0.64 was obtained, suggesting that a purifying selective pressure is acting on *pe_pgrs33* against deleterious SNPs. Among a total of 19 *pe_pgrs33* alleles identified in this study, 5 were cloned and used to complement the *pe_pgrs33* knock-out mutant strain of *Mtb* H37Rv (*Mtb*Δ33) to assess the functional impact of the respective polymorphisms in *in vitro* infections of primary macrophages. In human monocyte-derived macrophages (MDMs) infection, large in-frame and frameshift mutations were unable to restore the phenotype of *Mtb* H37Rv, impairing the cell entry capacity of *Mtb*, but neither its intracellular replication rate nor its immunomodulatory properties. *In vivo* studies performed in the murine model of tuberculosis (TB) demonstrated that the *Mtb*Δ33 mutant strain was not impaired in the ability to infect and replicate in the lung tissue compared to the parental strain. Interestingly, *Mtb*Δ33 showed an enhanced virulence during the chronic steps of infection compared to *Mtb* H37Rv. Similarly, the complementation of *Mtb*Δ33 with a frameshift allele also resulted in a *Mtb* strain capable of causing a surprisingly enhanced tissue damage in murine lungs, during the chronic steps of infection. Together, these results further support the role of PE_PGRS33 in the pathogenesis and virulence of *Mtb*.

## Introduction

*Mycobacterium tuberculosis* (*Mtb*), the main etiological agent of tuberculosis (TB) in humans, still represents one of the most feared pathogens at global level, with 10.4 million new cases of TB and 1.4 million deaths in 2015 (World Health Organization, 2016). According to recent comparative genomic studies, *Mtb* originated in Africa between 70,000 and 35,000 years ago from a genetic bottleneck of its progenitor (Gutierrez et al., 2005; Hershberg et al., 2008; Wirth et al., 2008), followed by the clonal expansion of 7 genetically homogeneous *Mtb* complex (MTBC) superlineages that ravaged human communities for centuries (Portevin et al., 2011; Gagneux, 2012; Firdessa et al., 2013).

The evolutionary scenario currently accepted for MTBC advocates the pathogen coevolution with different human populations and its association with specific geographical regions (Gagneux and Small, 2007; Hershberg et al., 2008; Wirth et al., 2008; Comas et al., 2013). Four MTBC superlineages were referred to as ancient and are mainly found in Eastern and Western Africa, South Eastern Asia and Southern India; the remaining three superlineages, denominated modern, are globally widespread and are responsible for the gravest TB epidemics in modern history (Gagneux, 2012; Firdessa et al., 2013). Despite the genetic homogeneity shared by MTBC superlineages compared to other bacterial pathogens, the presence of small sequence variations in MTBC genome is nevertheless responsible for differential pathogenetic properties (Gagneux and Small, 2007; Portevin et al., 2011).

It was hypothesized that most of the sequence variability among these genetically homogeneous bacteria rested on two large gene families, *pe* and *ppe*, covering approximately 7% of the *Mtb* genome coding capacity and encoding surface-exposed proteins (Cole et al., 1998; Banu et al., 2002; Brennan and Delogu, 2002; Mukhopadhyay and Balaji, 2011; Fishbein et al., 2015). The PE_PGRS and PPE_MPTR protein subfamilies, which are the most recently evolved within the respective families, are characterized by the presence of polymorphic regions at their C-terminus that vary in sequence and size (Gey van Pittius et al., 2006; McEvoy et al., 2012). *pe_pgrs* genes show GC-rich repetitive sequences (PGRS) which encode Gly-Gly-Ala/X repeats and *ppe_mptr* genes present polymorphic tandem repeats (MPTR) encoding Asn-(X-Gly)_2_-X-Asn-X-Gly repeats (Poulet and Cole, 1995; Sampson, 2011; Soldini et al., 2011; McEvoy et al., 2012).

One of the most investigated members of the *pe_pgrs* gene subfamily, the *pe_pgrs33* gene, was shown to be polymorphic among MTBC clinical strains, with SNPs and more frequently in-frame indels occurring in the PGRS domain of the protein and resulting in the gain/loss of one or more Gly-Gly-Ala/X repeats (Talarico et al., 2005, 2007; Wang et al., 2011; McEvoy et al., 2012). These findings provided an experimental support to the possible involvement of PE_PGRS33, as well as other PE_PGRS proteins, in the antigenic variability of *Mtb* (Talarico et al., 2005, 2007). Nevertheless, a recent study questioned this hypothesis in favor of the evolutionary conservation of the *pe_pgrs33* gene as indicated by the surprisingly low dN/dS ratio calculated taking into account 95 MTBC clinical strains (Copin et al., 2014). To date, *pe_pgrs33* alleles showing frameshift or large in-frame indels have been associated with non-cavitary pulmonary TB or extrapulmonary TB in children (Talarico et al., 2007; Wang et al., 2011), though the actual implications of these genetic variations on the pathogenesis and virulence of *Mtb* have not yet been explored.

In this study, we investigated for the first time the impact of polymorphisms occurring in *pe_pgrs33* alleles among *Mtb* clinical isolates in terms of pathogenesis and virulence, by exploiting complementation of the recently characterized *pe_pgrs33* mutant strain of *Mtb* H37Rv (*Mtb*Δ33) (Palucci et al., 2016). Based on previous evidences which implicated PE_PGRS33 in the pathogenesis of *Mtb* (Brennan et al., 2001; Palucci et al., 2016) and supported its role in mediating *Mtb* entry into macrophages and triggering of inflammatory responses in a TLR2-dependent mechanism (Brennan et al., 2001; Basu et al., 2007; Zumbo et al., 2013), here we assessed whether and how natural genetic variations may affect the functionality of PE_PGRS33 in *in vitro* and *in vivo* models of TB.

## Materials and methods

### MTBC strains, mycobacterial cultures and DNA extraction

One hundred thirty-five MTBC clinical strains were randomly selected from a collection of MTBC strains isolated at the Catholic University of the Sacred Heart in Rome between 2007 and 2011. Each clinical isolate was grown in Middlebrook 7H9 medium supplemented with 0.2% glycerol, 10% ADC and 0.05% Tween 80 at 37°C. Genomic DNA was extracted from liquid cultures by using the CTAB method, as previously described (van Embden et al., 1993).

### Molecular typing and phylogenetic analysis of MTBC strains

The genomic DNA of each MTBC clinical strain was genotyped by 15-loci MIRU-VNTR method (Gutierrez et al., 2005) and spoligotyping (Kamerbeek et al., 1997), as previously described. For MIRU-VNTR typing, each multiplex PCR set included the genome of *Mtb* H37Rv and water, as positive and negative controls, respectively. Similarly, in each experimental session of spoligotyping, both genomes of *Mtb* H37Rv and *M. bovis* BCG were used as positive controls and water as negative control. All multiplex PCRs for MIRU-VNTR typing were analyzed by capillary electrophoresis by using Applied Biosystems 3130xl Genetic Analyzer and PCR fragments size was estimated with the GeneMapper 4.0 software (Applied Biosystems). On MIRU-VNTR*plus* database, where possible, best-matches based on similarity search were inferred to the 15-loci MIRU-VNTR and spoligotyping patterns by setting a distance cut-off of 0.4, then a tree-based identification was performed by using default distance measures (www.miru-vntrplus.org; Gutierrez et al., 2005; Allix-Béguec et al., 2008). A UPGMA tree was eventually generated and rooted to the two reference strains of *M. canettii* available on the database.

### Sequencing of *pe_pgrs33* alleles and data analysis

The *pe_pgrs33* gene from each MTBC clinical strain was amplified with two primers previously described, PE_PGRS33-F1 and PE_PGRS33-R1 (Wang et al., 2011; Supplementary Table 1), by using the Expand High Fidelity PCR System (Roche) kit. Sanger sequencing was performed in Applied Biosystems 3130xl Genetic Analyzer. All identified polymorphisms were confirmed by double-strand DNA sequencing, as previously described (Talarico et al., 2005; Supplementary Table 1). Each DNA sequence was assembled respect to *pe_pgrs33* of *Mtb* H37Rv by using SeqMan (Lasergene 7). The genetic relationships between the 19 *pe_pgrs33* alleles identified in this study were investigated using MEGA version 6 (Tamura et al., 2013), by inferring a Maximum Likelihood tree under HKY model and setting 1000 bootstrap resampling. The selective pressure acting on all *pe_pgrs33* alleles was evaluated by calculating the dN/dS ratio with DnaSP (Rozas et al., 2003).

### Cloning of *pe_pgrs33* alleles and protein expression

Five of the 19 *pe_pgrs33* alleles identified in this study and their native promoters were amplified from the genome of one respective MTBC clinical isolate with two primers previously described, PG335Hn-338bp and 18c3AXb (Palucci et al., 2016; Supplementary Table 1), by using the Expand High Fidelity PCR System (Roche) kit. Each *pe_pgrs33* allele under the control of its native promoter was cloned into the integrative plasmid pMV306 in-frame with the HA epitope sequence (Palucci et al., 2016). The previously generated *Mtb*Δ33 mutant strain (Palucci et al., 2016) was then complemented with all constructs, according to procedures already described (Cascioferro et al., 2011; Palucci et al., 2016). To assess PE_PGRS33 expression, recombinant mycobacterial mid-log phase cells were homogenized in lysis buffer (10 mM Tris-HCl, 5 mM EDTA, protease inhibitors cocktail, pH 9.5) and 0.1 mm zirconia/silica beads (Biospec Products), as previously described (De Maio et al., 2014). Protein lysates, devoid of cellular debris and unlysed cells, were separated by SDS-PAGE on 12% polyacrylamide gels and were then transferred to nitrocellulose membrane by western blot. HA-tagged PE_PGRS33 was detected by using anti-HA monoclonal antibody HA.11 (1:1,000) (Covance), as primary antibody, and anti-mouse IgG peroxidase conjugate (1:4,000) (Sigma-Aldrich, Saint Louis, MO), as secondary antibody. Since, as expected, the protein encoded by 33^all3^ was undetectable by western blot, two control PCRs were carried out by using the genomic DNA of *Mtb* H37Rv and the pMV306 plasmid engineered with the 33^all3^ allele under the control of its native promoter (subsequently used to complement the *Mtb*Δ33 mutant) as positive controls and water as negative control (Supplementary Figure 1; Supplementary Table 1). As further control, polymorphisms in 33^all3^ of the *Mtb*Δ33::33^all3^ strain were reconfirmed by Sanger sequencing (data not shown).

### Cell cultures and *in vitro* infections

Mice manipulation was performed prior approval by the Ethics Committee of the Catholic University of the Sacred Heart in Rome (Prot. Number: n° T21/2011). Peritoneal murine macrophages (pMMOs) were isolated from 9 to 14 weeks old female C57BL/6 mice (Harlan), as previously described (Palucci et al., 2016). pMMOs were seeded at 1.2·10^6^cells/ml in 48-well plates in RPMI medium supplemented with 10% FBS and 1% of both L-glutamine and sodium pyruvate and were incubated overnight at 37°C in a 5% humidified atmosphere. After removing non-adherent cells, pMMOs were infected for 1 h with *Mtb* strains at a multiplicity of infection (MOI) of 1:10 and then incubated in complete RPMI medium. Four hours later, intracellular mycobacteria were determined by CFUs enumeration, as previously described (Palucci et al., 2016). Buffy coats of 4 male donors were processed to collect human peripheral blood mononuclear cells (PBMCs) and monocyte-derived macrophages (MDMs). PBMCs were isolated from buffy coats by using Ficoll, seeded for mycobacterial infections at 1.2·10^6^ cells/ml in 48-well plates in X-VIVO™ 15 medium (Lonza) with human serum type AB 2% (Lonza) and incubated overnight at 37°C in a 5% humidified atmosphere. MDMs were isolated by positive selection from unplated PBMCs by using CD14 MicroBeads, following the manufacturer's instructions (Miltenyi Biotec). MDMs were seeded at 1.2·10^6^ cells/ml in 48-well plates, as indicated for PBMCs and were incubated for 6–7 days at 37°C in a 5% humidified atmosphere until MDM differentiation. Both PBMCs and MDMs were infected with *Mtb* strains at a MOI of 1:1. According to the method described above for pMMOs, MDMs were infected and intracellular mycobacterial CFUs were determined at 4 and 72 h post-infection (Palucci et al., 2016). For PBMCs infections, mycobacterial *inocula* at a MOI of 1:1 were directly added to cell culture supernatants and at 72 h post-infection total mycobacterial CFUs were enumerated (Palucci et al., 2016). Specifically, cell pellets obtained prior centrifugation of supernatants from PBMCs infection were lysed and resuspended in PBS with 0.05% Tween 80, where MDMs lysed in 0.1% Triton X-100 were previously added, then total lysates were serially diluted and plated on 7H11/OADC agar medium (Palucci et al., 2016). Supernatants from PBMCs and MDMs at 72 h post-infection were collected and stored a −80°C until being assayed for cytokine ELISA.

### Cytokine analysis

Supernatants collected from MDMs and PBMCs at 72 h post-infection were filtered to remove mycobacteria and cytokines were analyzed by using a Cytometric Bead Array (CBA) (BD Biosciences, San Jose, USA) and a FACS CANTO II (BD Biosciences, San Jose, USA), according to the manufacturer's instructions. CBA results were generated by FCAP Array™ software (BD Biosciences, San Jose, USA). For supernatants collected from PBMCs at 72 h post-infection, IFN-γ release was also evaluated by using ELISA QuantiFERON TB-Gold (Qiagen, Hilden, Germany), according to the manufacturer's instructions.

### *In vivo* infection

Animal studies were carried out in strict accordance with the Amsterdam protocol on animal protection and welfare, the Directive 2010/63/EU of the European Parliament and the Council of 22 September 2010 on the protection of animals used for scientific purposes and the French Decree 2013-118. The protocol was approved by the Minister of Higher Education and Research after favorable opinion of the Ethics Committee (CEEA Nord-Pas de Calais/INSERMU1019 n° 00579.01 from 23/07/2014). All efforts were made to minimize suffering of the animals. Six-weeks-old female BALB/c mice (Janvier) were challenged with mycobacterial strains *via* the intranasal route with 10 μl/nostril. Challenge suspensions were adjusted in order to obtain an inhaled dose of approximately 1,000 CFU/lungs. At day 0, 28, and 49 post-infection, lungs from euthanized mice were collected and homogenized by using an MM300 apparatus (Qiagen) and 2.5-mm diameter glass beads. Ten-fold serial dilutions of lung homogenates were plated on 7H11/OADC agar medium and CFUs enumerated, as previously described (Palucci et al., 2016).

### Histopathological analysis

Murine lungs were fixed with 10% paraformaldehyde and then embedded in paraffin for sectioning, according to standard methods. In order to correlate the presence of histologic lesions with acid-fast bacilli (AFB), replicas 3 μm sections were cut and stained with both Haematoxyline and Eosin (HE) and Ziehl–Neelsen (ZN), following standard techniques. The lesions morphology and distribution were evaluated by light microscopy. At least 6 lung sections for all mice of the 5 groups were analyzed in different points (24 lung sections *per* experimental group in total). For each section the number of ZN positive cells, the total surface area and the area with lesions were measured at 400x magnification and averages calculated for each section and group. Slides were imaged using Nikon Eclipse 80i microscope and digital computer images were recorded with a Nikon DS-L2 camera control unit and the Nikon dedicated software 3422.1001.1798.080117. Cellular automatic count and histological measurements were carried out using the dedicated software Axiovision ver. 4.4 (Zeiss) by two independent researchers on two independent photo series.

### Immunological colocalization

This assay was carried out by using two monoclonal antibodies (mAb), purified biotin anti-F4/80 Clone BM8 (Caltag Labs, cat. n. MF48000) and purified anti-MT 16 kDa antigen (Santa Cruz Biotechnology, cat. n. sc-58169). Signals were revealed with Streptavidin Alexa Fluor® 555 and Streptavidin Alexa Fluor® 488. For immunomicroscopy, slides obtained from fixed-lung tissues were also processed, as previously described, and mounted on positively charged Superfrost slides (Fisher Scientific). Deparaffinization, rehydration and antigens retrieval of tissue sections were performed by using Dewax and HIER Buffer L (Thermo Fisher Scientific). To prevent non-specific bindings, slides were incubated in PBS containing 2% BSA, stabilizing protein and 0.015 mol/L sodium azide (Protein Block Serum-Free, Dako). For each target, tissue sections were incubated in two different steps with primary mAb (overnight at 4°C), Ab (1 h at room temperature) and the respective fluorophore. Slides were counterstained with Hoechst blue and then covered. Images were acquired by using a Leica TCS SP 5 confocal microscope (Leica Microsystems, Germany) and processed with LAS AF Lite application software developed by Leica Microsystems CMS GmbH for contrast and brightness adjustments. Negative controls prepared by omission of primary antibodies did not show any fluorescence under the conditions described above.

### Statistical analysis

Experiments were conducted in triplicate and replicated at least three times. *In vivo* experiment was performed one time in quintuplicate. Statistical analysis was performed by using GraphPad Prism version 6 (GraphPad software, CA, USA). For pMMOs infections, CFUs were expressed as mean ± SD and analyzed by one-way ANOVA, followed by Dunnett's multiple comparison test. For infections of human PBMCs and MDMs, results concerning CFUs and cytokine levels were expressed as median and analyzed by Kruskal-Wallis one-way ANOVA, followed by Dunnett's multiple comparison test. Results obtained from *in vivo* experiments were expressed as mean ± SD and analyzed by performing two-way ANOVA, followed by Dunnett's multiple comparison test. Histopathological results were analyzed by Student's *t*-test.

## Results

### Superlineage-based composition and phylogeny of 135 MTBC clinical strains collected in rome

**Figure 1:**
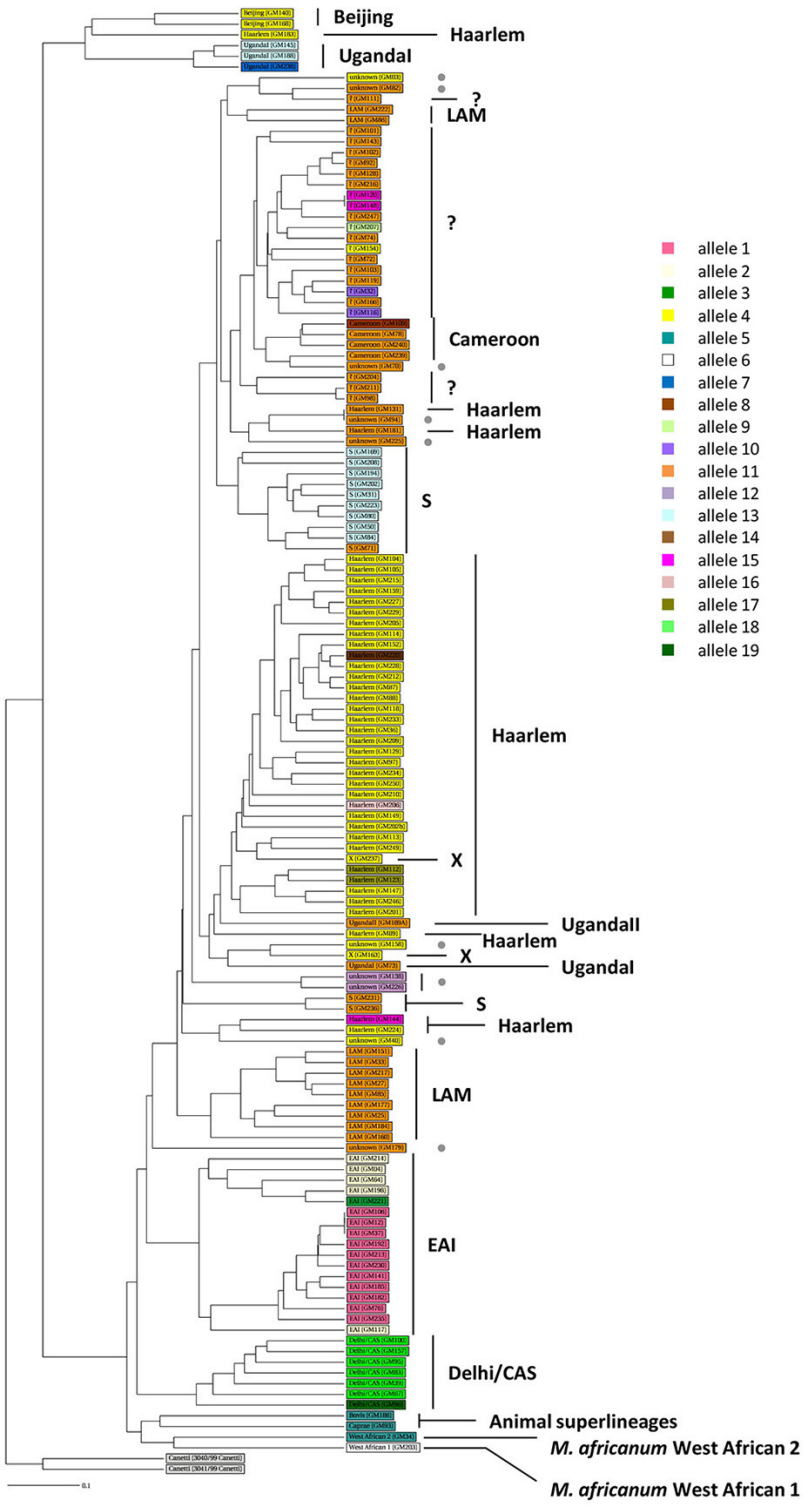
**Phylogeny of 135 MTBC clinical strains color-coded by the *pe_pgrs33* alleles**. One hundred thirty-five MTBC clinical strains isolated in Rome were genotyped by 15 loci MIRU-VNTR (Gutierrez et al., 2005) combined with spoligotyping (Kamerbeek et al., 1997). An UPGMA tree rooted by two previously characterized strains of *M. canetti* was obtained from MIRU-VNTR*plus* database (Gutierrez et al., 2005; Allix-Béguec et al., 2008) and a different color-coded by the *pe_pgrs33* alleles was assigned. Gray dot, unknown strains; “?,” strains likely belonging to the T-specific lineage of the Euro-American superlineage (Allix-Béguec et al., 2008).

One hundred thirty-five MTBC clinical strains isolated in Rome from 2007 to 2011 were genotyped by using 15-loci MIRU-VNTR (Gutierrez et al., 2005) combined with spoligotyping (Kamerbeek et al., 1997). The *in silico* analysis performed on MIRU-VNTR*plus* database (Gutierrez et al., 2005; Allix-Béguec et al., 2008) allowed to identify 125 isolates (92.6%) and assign the respective MTBC superlineage (Supplementary Figure 2). The majority of isolates (104 isolates, 77%) belonged to the modern MTBC superlineages, which comprised the superlineages 2 (East-Asian, 2 isolates, 1.5%), 3 (East-African Indian, 7 isolates, 5.2%) and 4 (Euro-American, 95 isolates, 70.4%). This latter was composed of the Cameroon (4 isolates, 4.2%), Haarlem (39 isolates, 41.1%), LAM (11 isolates, 11.6%), S (12 isolates, 12.6%), Uganda I (4 isolates, 4.2%), Uganda II (1 isolate, 1.1%), X (2 isolates, 2.1%) lineages and unassigned strains (22 isolates, 23.2%, indicated in Supplementary Figure 2 as “?”) which could plausibly belong to the T-specific lineage of the Euro-American superlineage (Allix-Béguec et al., 2008). Although to a lesser extent (21 isolates, 15.6%), our collection of MTBC strains embraced three ancient superlineages: the most representative was the superlineage 1 (Indo Oceanic, 17 isolates, 12.6%), followed by the superlineages 5 and 6 (West African 1 and West African 2, respectively, 1 isolate, 0.7% each) *plus* the animal superlineage (2 isolates, 1.5%). The phylogenetic relationships between all 135 genotyped MTBC clinical strains were inferred by constructing an UPGMA tree rooted by two reference strains of *M. canettii* on MIRU-VNTR*plus* database (Figure 1; Gutierrez et al., 2005; Allix-Béguec et al., 2008). According to the phylogeographical distribution described for the MTBC by Gagneux and colleagues (Gagneux, 2012), our results showed how all MTBC superlineages are represented in the metropolitan area of Rome, except for the recently described ancient superlineage denominated 7 or Ethiopian (Firdessa et al., 2013), and that the Euro-American superlineage is the most prevalent.

### MTBC clinical strains and *pe_pgrs33* alleles association

The genetic variability of the *pe_pgrs33* gene within the MTBC population analyzed in this study was assessed by Sanger sequencing. As shown in Supplementary Table 2, a total of 19 *pe_pgrs33* alleles were identified. Apart from the allele referred to as 11, which corresponded to *pe_pgrs33* of *Mtb* H37Rv and was found in 42 MTBC clinical isolates (Figure 1), we identified 11 alleles previously described in literature (Talarico et al., 2005, 2007; Wang et al., 2011; McEvoy et al., 2012) and 7 alleles containing new genetic variations (alleles 3, 7, 8, 9, 14, 16, and 17) compared to *pe_pgrs33* of the H37Rv reference strain. Among all *pe_pgrs33* alleles, we identified a total of 13 SNPs (9 non-synonymous and 4 synonymous), mainly occurring in the PGRS region. All indel events were exclusively located in the PGRS domain, the majority of which were in-frame and varied from a minimum of 9 bp to a maximum of 72 bp. The largest mutations were in-frame deletions found in 11 EAI isolates (allele 1), in the only isolate of *M. africanum* belonging to the West African 1 superlineage (allele 6) and in all the 7 Delhi/CAS isolates (alleles 18 and 19) (Figure 1). The only frameshift mutation identified was 1 bp deletion in position 1,014 bp and was detected in 3 *pe_pgrs33* alleles (alleles 1, 2, and 3) all belonging to the EAI superlineage (Figure 1). Specifically, the altered frame in these latter alleles results in the introduction of a premature stop codon, which translates into the loss of the last 160 amino acids from the C-terminal of the PGRS domain and the “acquisition” of a 36-amino acids stretch, rich in proline residues (approximately 20%) and short antigenic epitopes, as revealed by the *in silico* antigenicity prediction of the protein (Supplementary Figure 3). The 3 *pe_pgrs33* alleles, sharing the only frameshift mutation identified in this study, were more evolutionarily distant from the other alleles in the unrooted tree shown in Supplementary Figure 4. Moreover, a strict association between these alleles and the EAI superlineage was revealed by the 15-loci MIRU-VNTR and spoligotyping-based UPGMA tree color-coded by the *pe_pgrs33* alleles (Figure 1). Similarly, a “clustered” association within the MTBC superlineages was quite fulfilled in the same tree also for the other alleles. In agreement with the results of Copin et al. (2014), the dN/dS ratio of 0.64 obtained for the *pe_pgrs33* alleles identified in this study supports the evidence that a purifying selection is acting on *pe_pgrs33* to preserve the gene sequence from deleterious SNPs.

### *pe_pgrs33* alleles and protein expression

**Figure 2:**
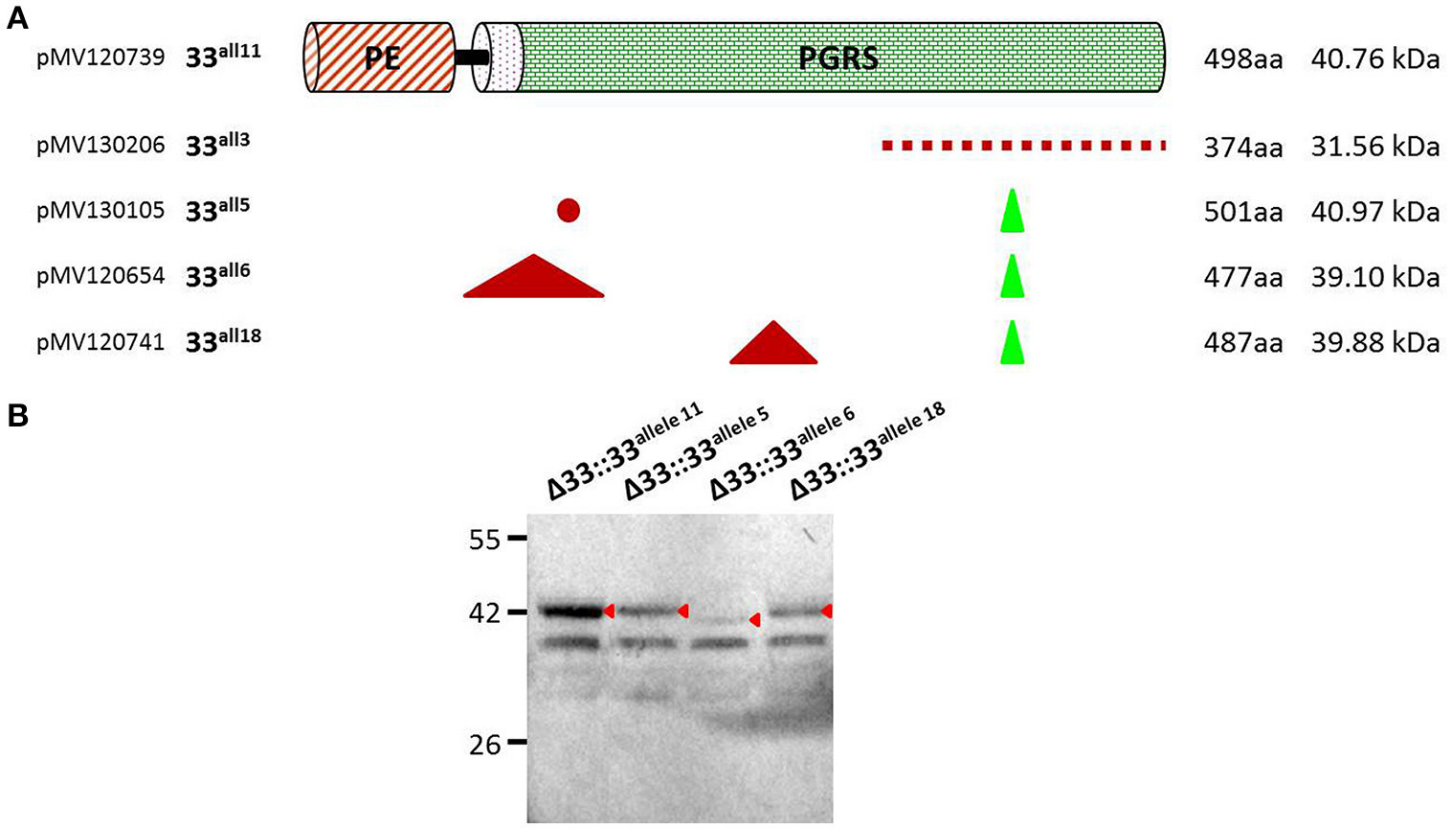
**Schematic and details of amino acid variations in the 5 *pe_pgrs33* alleles-encoded proteins selected in this study**. Five *pe_pgrs33* alleles under the control of the respective native promoter were amplified and cloned upstream the HA epitope sequence into the integrative plasmid pMV306 (Palucci et al., 2016). **(A)** Upper in the cartoon is schematized the protein encoded by 33^all11^ without natural mutations respect to PE_PGRS33 of *Mtb* H37Rv. Amino acid variations occurring in the proteins encoded by 33^all3^, 33^all5^, 33^all6^, and 33^all18^ are indicated with different symbols (red dashed line, different amino acid composition due to a frameshift mutation in the respective gene; red dot, single amino acid substitution; green triangle, amino acids inserted; red triangle, amino acids deleted). Cloning and protein details are reported beside. **(B)** Protein lysates of the *Mtb*Δ33 complemented strains were analyzed by western blot. Due to the frameshift deletion, the protein encoded by 33^all3^ was undetectable with an anti-HA antibody and was thus not included in this analysis (cfr. Supplementary Figure 1 for control PCRs).

Until recently, a possible role of PE_PGRS33 in the antigenic variability of *Mtb* has been entrenched with the identification of numerous polymorphisms in the *pe_pgrs33* gene among *Mtb* clinical isolates (Talarico et al., 2005, 2007; Wang et al., 2011; McEvoy et al., 2012). However, a recent study questioned this hypothesis, suggesting an ongoing purifying selection on *pe_pgrs33* and a limited impact on the functionality and antigenicity of PE_PGRS by indel events occurring in the PGRS domain (Copin et al., 2014). To elucidate the biological meaning of natural polymorphisms occurring in *pe_pgrs33* and their impact on the pathogenesis and virulence of *Mtb*, 5 of the 19 *pe_pgrs33* alleles identified in this study, designated from here as 33^allx^ (where “x” referred to the number of the respective allele), were selected based on the heterogeneity of their genetic variations. Among the selected alleles, 4 (33^all3^, 33^all5^, 33^all6^, and 33^all18^) were characterized by SNPs, in-frame indels and a single frameshift deletion occurring in the region encoding the PGRS domain of PE_PGRS33 compared to the 33^all11^ allele, which corresponds to *pe_pgrs33* of *Mtb* H37Rv (Table 1; Figure 2A). Moreover, no polymorphisms were detected in the native promoter sequence of the 5 *pe_pgrs33* alleles used in this study. These alleles were cloned in the integrative plasmid pMV306, under the control of their native promoter and upstream the HA epitope sequence, and all plasmids used to complement the previously generated *Mtb*Δ33 mutant strain (Palucci et al., 2016; Figure 2). Transcriptional analysis by RT real-time PCR was used to confirm expression in the complemented strains and higher expression of *pe_pgrs33* was observed in the complemented strains compared to the wild type (+2,5-fold, data not shown), as expected when the gene is inserted in the *att*B site following complementation with the integrative plasmid pMV306 (Palucci et al., 2016). Apart from *Mtb*Δ33::33^all3^, for all complemented strains, PE_PGRS33 expression was assessed on protein lysates by western blot using an anti-HA antibody (Figure 2B). For *Mtb*Δ33::33^all3^, PCR (Supplementary Figure 1) and Sanger sequencing (data not shown) allowed to confirm both strain complementation and the altered frame in 33^all3^.

**Table 1:** **Details of genetic variations for the 5 *pe_pgrs33* alleles selected in this study**.

***pe_pgrs33*** **allele**	**Genetic variation**	**Position (bp)**
3	sSNP	582
	sSNP	717
	−1 bp	1,014
	(+9 bp)	(1,240)
5	nsSNP	697
	sSNP	717
	+9bp	1,240
6	−72 bp	416–487
	sSNP	717
	+9 bp	1,240
11	None*^a^*
18	sSNP	717
	−42 bp	772–813
	+9 bp	1,240

a *None compared to pe_pgrs33 gene of Mtb H37Rv (NC_000962.3). Each color has been assigned to distinguish different alleles*.

### The altered frame of *pe_pgrs33* negatively affects the protein-mediated entry of *Mtb* into murine macrophages

**Figure 3:**
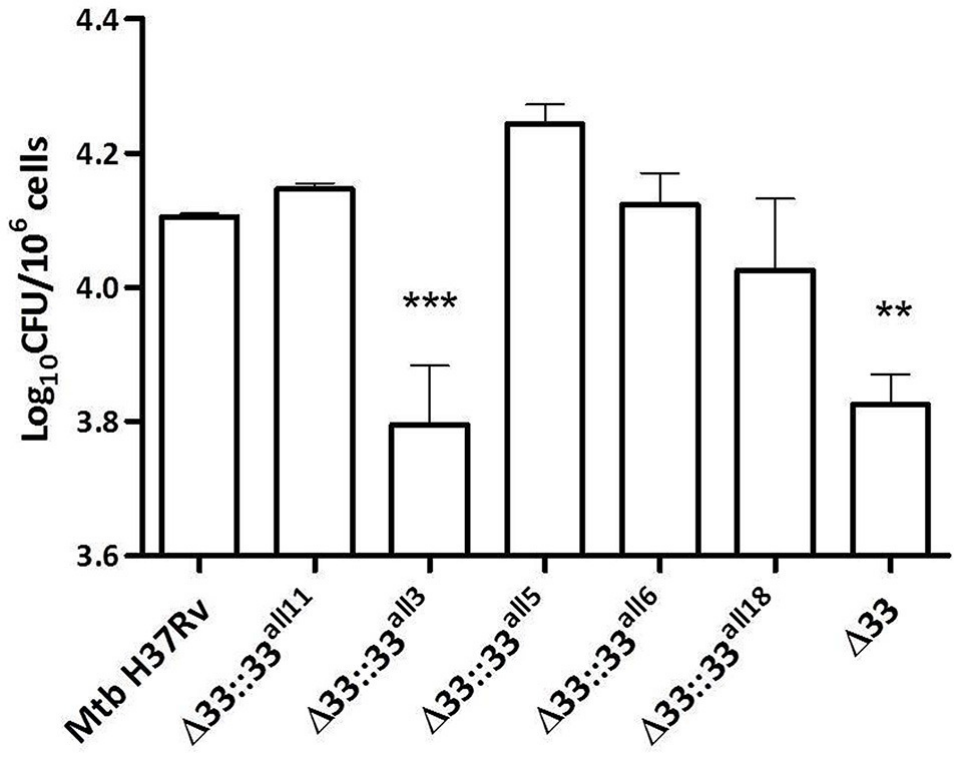
**Frameshift mutation in *pe_pgrs33* negatively affects the protein-mediated entry of *Mtb* into murine macrophages**. pMMOs were infected at a MOI of 1:10 with *Mtb* H37Rv, the *Mtb*Δ33 mutant and a panel of complemented strains (*Mtb*Δ33::33^all11^, *Mtb*Δ33::33^all3^, *Mtb*Δ33::33^all5^, *Mtb*Δ33::33^all6^, and *Mtb*Δ33::33^all18^). For all *Mtb* strains, mean value of CFUs in triplicate from a representative experiment of pMMOs infection and the respective standard deviation are represented. ^**^p < 0.01 and ^***^p < 0.001 compared to *Mtb* H37Rv (one-way ANOVA followed by Dunnett's multiple comparison test).

In previous studies, large in-frame sequence variations in the *pe_pgrs33* gene were associated to peculiar epidemiological phenotypes, like non-cavitary pulmonary TB or extrapulmonary TB in children (Talarico et al., 2005, 2007; Wang et al., 2011). To investigate the pathogenic impact of large sequence variations occurring in *pe_pgrs33* among MTBC clinical isolates, peritoneal murine macrophages (pMMOs) were infected at a MOI 1:10 with the *Mtb* H37Rv, *Mtb*Δ33 mutant and complemented strains with all 5 *pe_pgrs33* alleles selected in this study (*Mtb*Δ33::33^all11^, *Mtb*Δ33::33^all3^, *Mtb*Δ33::33^all5^, *Mtb*Δ33::33^all6^, and *Mtb*Δ33::33^all18^). Since we recently showed that the *Mtb*Δ33 mutant is impaired in its ability to enter into macrophages (Palucci et al., 2016), the capacity to restore this phenotype by the complemented strains was assessed at 4 h post-infection by enumerating intracellular CFUs (Figure 3). Similar to the *Mtb*Δ33::33^all11^ strain, *Mtb*Δ33::33^all5^, *Mtb*Δ33::33^all6^, and *Mtb*Δ33::33^all18^ were able to entry into pMMOs as efficiently as the parental strain. The significant reduction in this capacity for the *Mtb*Δ33 mutant (*p* < 0.01) was also markedly observed for *Mtb*Δ33::33^all3^ (*p* < 0.001), indicating that whilst frameshift mutation in *pe_pgrs33* alleles impair the cell entry phenotype of *Mtb*, small and large in-frame sequence variations as well as nsSNPs do not affect the PE_PGRS33-mediated entrance of *Mtb* into murine macrophages.

### Large sequence variations occurring in *pe_pgrs33* alleles impair *Mtb* entrance, but neither replication nor immunomodulation during infection of human primary cells

**Figure 4:**
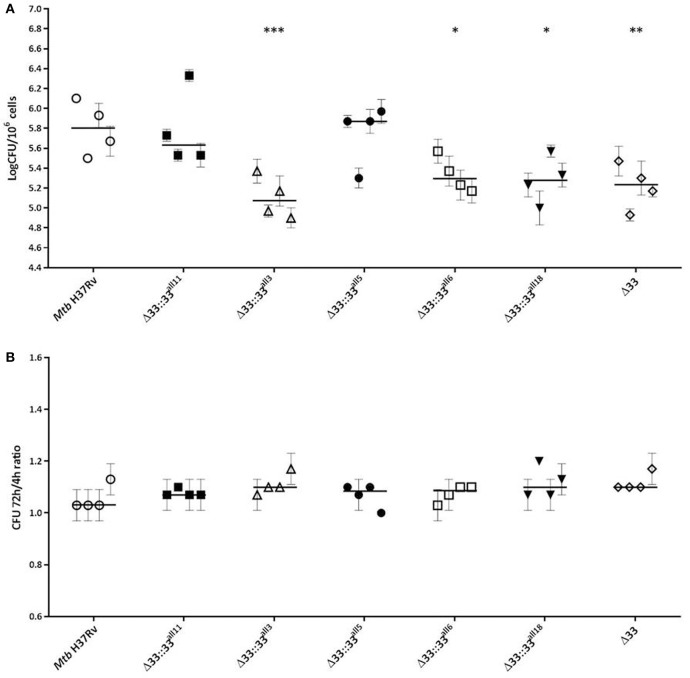
**Large natural mutations in PE_PGRS33 impair entry, but do not affect the replication phenotype of *Mtb* H37Rv in *in vitro* human MDMs infections**. Human MDMs were infected at a MOI of 1:1 with *Mtb* H37Rv, the *Mtb*Δ33 mutant and complemented strains (*Mtb*Δ33::33^all11^, *Mtb*Δ33::33^all3^, *Mtb*Δ33::33^all5^, *Mtb*Δ33::33^all6^, and *Mtb*Δ33::33^all18^). Mycobacterial entry and replication phenotypes were assessed by CFUs enumeration of intracellular mycobacteria at 4 h **(A)** and 72 h post-infection, respectively. Logarithmic scale of CFUs ratio between intracellular mycobacteria at 72 and 4 h post-infection **(B)**. For all *Mtb* strains, mean value of CFUs in triplicate from MDMs infections of 4 donors and the respective standard deviation are represented. Bars indicate the median. ^*^p < 0.05, ^**^p < 0.01, and ^***^p < 0.001 compared to *Mtb* H37Rv (Kruskal-Wallis one-way ANOVA followed by Dunnett's multiple comparison test).

**Figure 5:**
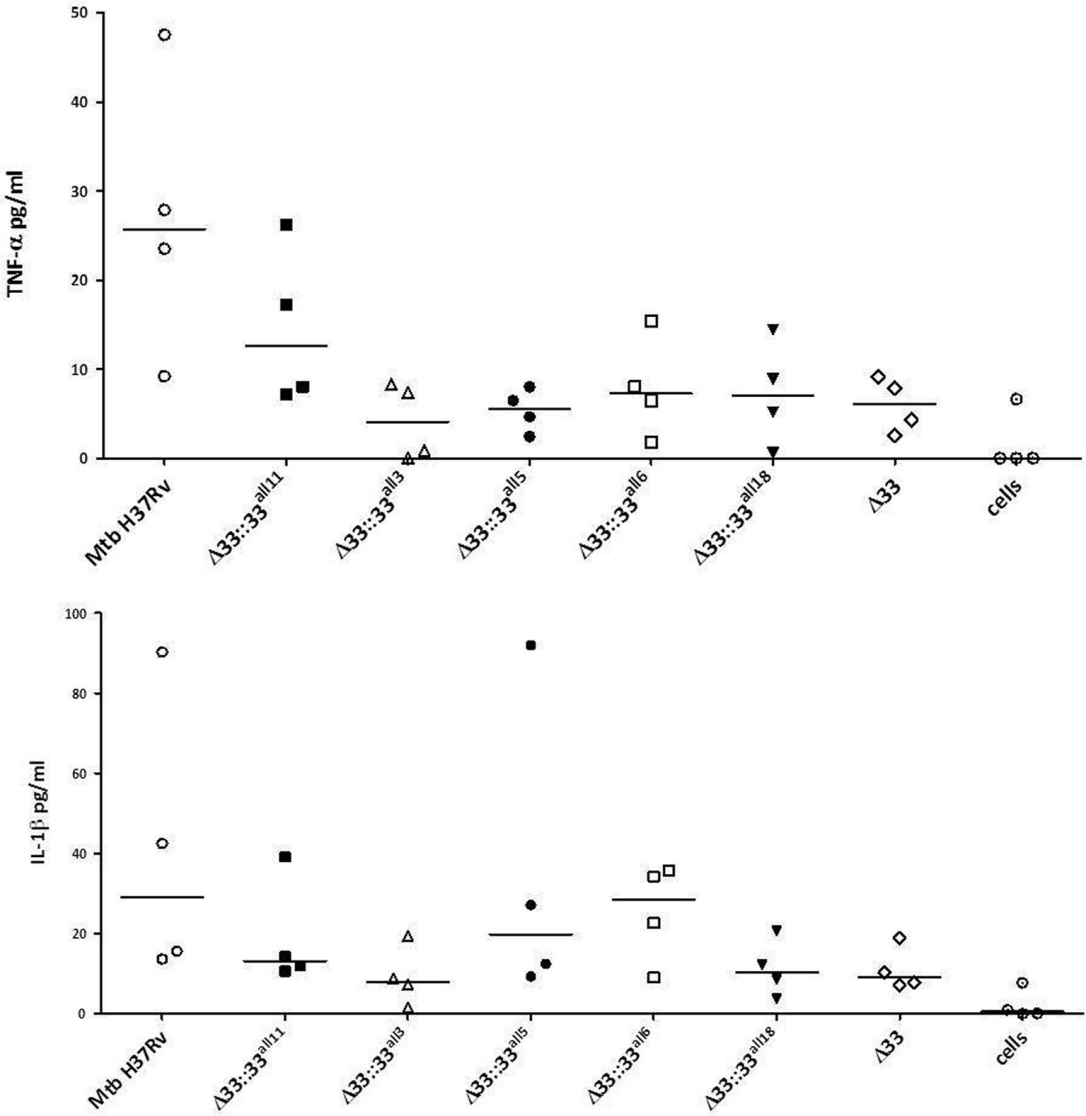
**Polymorphisms in *pe_pgr33* do not affect the immunomodulatory properties of the protein in *in vitro* human MDMs infections**. Supernatants collected at 72 h post-infection were assessed for TNF-α and IL-1β. For all *Mtb* strains, each symbol corresponds to the mean of triplicates obtained from MDMs infections of 4 healthy donors. Bars indicate the median. None statistically significant difference was observed compared to *Mtb* H37Rv (Kruskal-Wallis one-way ANOVA followed by Dunnett's multiple comparison test).

**Figure 6:**
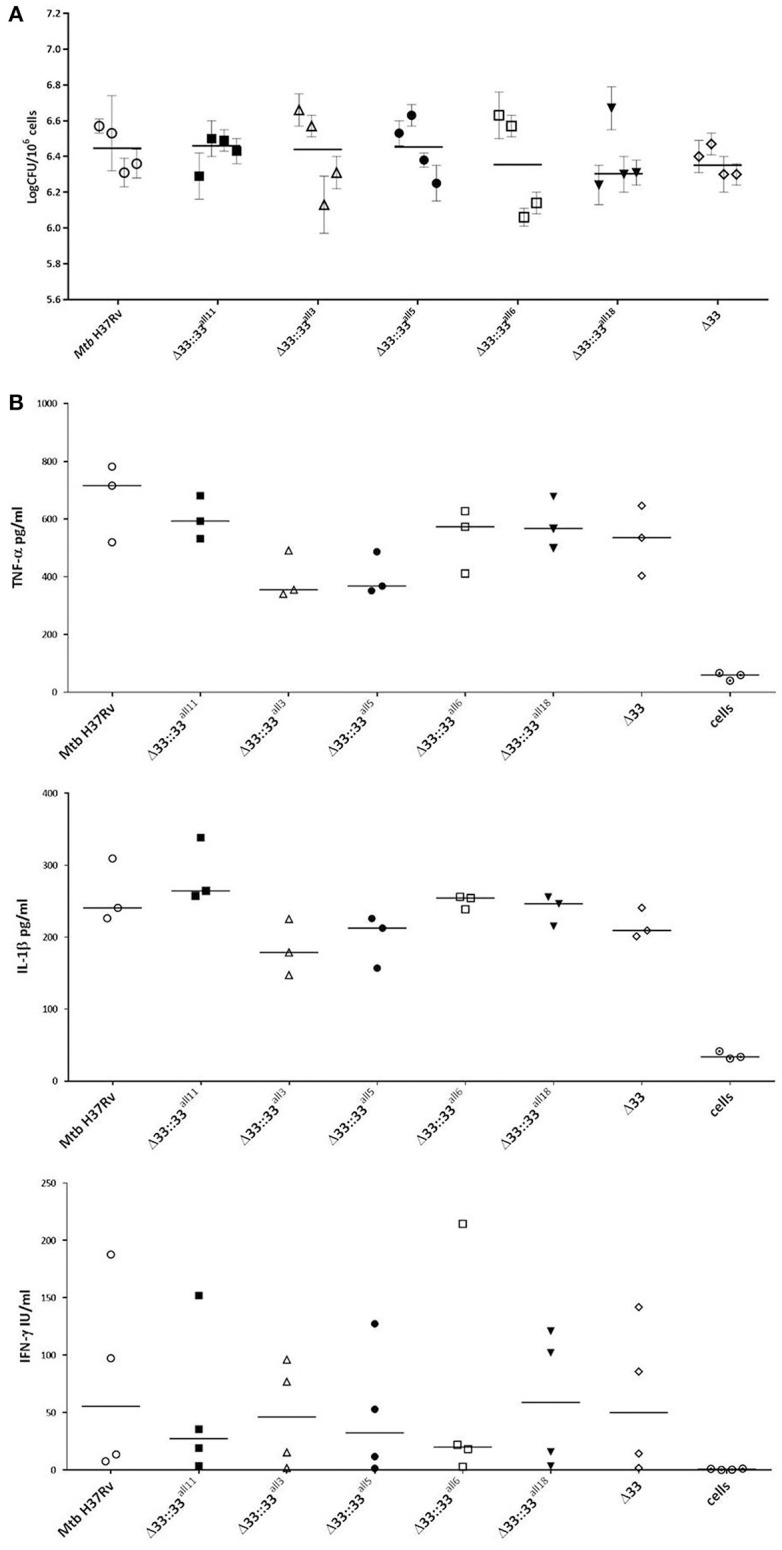
***Mtb*** **replication and immunomodulation is not impaired by *pe_pgrs33* polymorphisms in PBMCs infections**. PBMCs were infected at a MOI of 1:1 with *Mtb* H37Rv, the *Mtb*Δ33 mutant and complemented strains (*Mtb*Δ33::33^all11^, *Mtb*Δ33::33^all3^, *Mtb*Δ33::33^all5^, *Mtb*Δ33::33^all6^, and *Mtb*Δ33::33^all18^). Mycobacterial replication was assessed by CFUs enumeration of total mycobacteria at 72 h post-infection **(A)**. For each *Mtb* strain, mean value of CFUs obtained in triplicate from PBMCs infection of all 4 donors and the respective standard deviation are reported. Bars indicate the median. Supernatants collected at 72 h post-infection were assessed for TNF-α, IL-1β, and IFN-γ **(B)**. For all *Mtb* strains, each symbol corresponds to the mean of triplicates obtained from PBMCs infections of 3 or 4 healthy donors, depending on the cytokine panel. Bars indicate the median. For both CFUs and cytokine analysis, none statistically significant difference was observed compared to *Mtb* H37Rv (Kruskal-Wallis one-way ANOVA followed by Dunnett's multiple comparison test).

The impact of *pe_pgrs33* polymorphisms was further investigated *in vitro* by infecting human monocyte-derived macrophages (MDMs) isolated from healthy donors with the *Mtb* H37Rv, *Mtb*Δ33 mutant and complemented strains at a MOI 1:1 (Figure 4). *Mtb*Δ33::33^all11^ and *Mtb*Δ33::33^all5^ were able to complement the entry phenotype into MDMs compared to the parental strain (Figure 4A). Similar to the *Mtb*Δ33 mutant (*p* < 0.01), *Mtb*Δ33::33^all3^ (*p* < 0.001), *Mtb*Δ33::33^all6^ (*p* < 0.05), and *Mtb*Δ33::33^all18^ (*p* < 0.05) were unable to fully restore the ability of *Mtb* H37Rv to entry into macrophages. To assess the impact of *pe_pgrs33* polymorphisms on the capacity of *Mtb* to replicate intracellularly, macrophages infected as above were harvested at 72 h to determine CFUs and results expressed as logarithmic ratio between intracellular CFUs at 72 and 4 h. As shown in (Figure 4B), no statistically significant differences were observed for the *Mtb*Δ33 mutant and complemented strains compared to *Mtb* H37Rv. Based on previous evidences on the immune modulating properties of PE_PGRS33 (Basu et al., 2007; Zumbo et al., 2013), two pro-inflammatory cytokines, TNF-α and IL-1β, were evaluated by performing a cytometric bead array (CBA) assay on supernatants collected at 72 h from MDMs infection. In (Figure 5), results of TNF-α and IL-1β levels are reported and show a reduced ability for the *Mtb*Δ33 mutant and complemented strains to elicit TNF-α secretion compared to *Mtb* H37Rv, even if this difference did not reach statistical significance. To further assess whether *pe_pgrs33* genetic polymorphisms could elicit peculiar phenotypes during infection of human primary cells, in terms of replication capacity of *Mtb* and modulation of host immune responses by the pathogen, we used the panel of selected mycobacterial strains to infect peripheral blood mononuclear cells (PBMC) isolated from the same donors. Also in this cellular model, mycobacterial CFUs enumerated at 72 h post-infection did not result in significant differences in the replication capacity of all recombinant *Mtb* strains compared to *Mtb* H37Rv (Figure 6A). Moreover, no significant perturbations in the ability of *Mtb* to induce a TNF-α, IL-1β, and IFN-γ-mediated proinflammatory response were detected in PBMCs infections, although *Mtb*Δ33::33^all3^ and *Mtb*Δ33::33^all5^ showed a slight decreased capacity to induce both TNF-α, and IL-1β (Figure 6B) secretion in line with what observed in MDMs infections.

### Major genetic variations in *pe_pgrs33* differently affect *Mtb* replication during the chronic persistent TB disease stages in murine infection model

**Figure 7:**
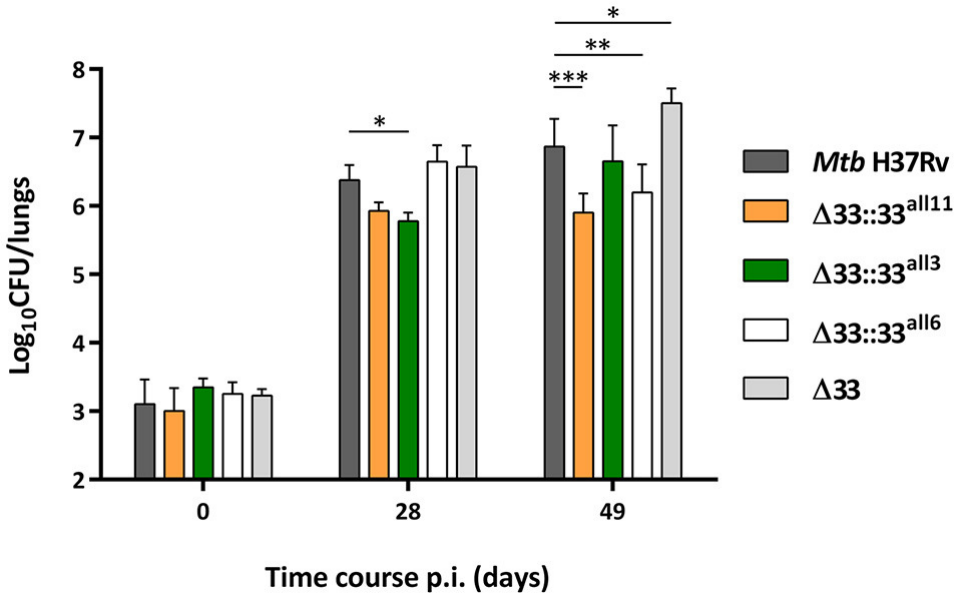
**Large natural mutations in the *pe_pgrs33* gene differently affect the replication capacity of *Mtb* in murine lungs**. To evaluate the lung colonization and replication capacity of *Mtb*Δ33 mutant strains complemented with *pe_pgrs33* alleles characterized by large genetic variations (frameshift and in-frame deletions), 5 groups of BALB/c mice (4 mice *per* group) were challenged *via* intranasal route with *Mtb* H37Rv, the *Mtb*Δ33 mutant and complemented strains (*Mtb*Δ33::33^all11^, *Mtb*Δ33::33^all3^, and *Mtb*Δ33::33^all6^). CFUs in murine lungs were then enumerated at day 0, 28, and 49 post-infection. For all *Mtb* strains, mean value of CFUs in quadruplicate from a representative experiment of mice infection and the respective standard deviation are represented. ^*^p < 0.05, ^**^p < 0.01, and ^***^p < 0.001 (two-way ANOVA followed by Dunnett's multiple comparison test).

To assess the role of PE_PGRS33 on the virulence of *Mtb*, 5 groups of BALB/c mice were intranasally infected with *Mtb* H37Rv, the *Mtb*Δ33 mutant and 3 complemented strains selected from those previously assessed *in vitro*, one expressing the allele corresponding to *pe_pgrs33* of *Mtb* H37Rv (*Mtb*Δ33::33^all11^) and two expressing *pe_pgrs33* alleles characterized by major genetic variations (*Mtb*Δ33::33^all3^ and *Mtb*Δ33::33^all6^) (Figure 7). At day 0 post-infection, the number of CFUs enumerated in murine lungs did not significantly vary, indicating that the *Mtb* H37Rv, *Mtb*Δ33 and complemented strains were similarly able to infect mice. At day 28 post-infection, which is considered the hallmark of active disease in mice, no variation of the bacterial burden in the lungs was found for both *Mtb*Δ33::33^all6^ and *Mtb*Δ33 compared to the parental strain. Conversely, a reduced bacterial load was detected for *Mtb*Δ33::33^all11^ and for *Mtb*Δ33::33^all3^ strains compared to *Mtb* H37Rv. Interestingly, at day 49 post-infection, lung CFUs from mice infected with *Mtb*Δ33 were higher (*p* < 0.05) than those from mice infected with *Mtb* H37Rv and increased significantly (*p* < 0.001) from the CFUs enumerated at day 28 post-infection, indicating that the lack of PE_PGRS33 results in an enhanced ability of *Mtb* to replicate/persist in the lung tissue of mice during the chronic phases of the disease. A similar trend was also found for *Mtb*Δ33::33^all3^, with lung CFUs significantly higher at day 49 compared to day 28 post-infection (*p* < 0.01). Conversely, bacterial loads were significantly reduced for *Mtb*Δ33::33^all11^ (*p* < 0.001) and *Mtb*Δ33::33^all6^ (*p* < 0.01) compared to the parental strain at day 49 post-infection.

### The lack and truncation of PE_PGRS33 in *Mtb* is responsible for the extent of tissue damage in murine lungs during the chronic persistent TB disease stages

**Figure 8:**
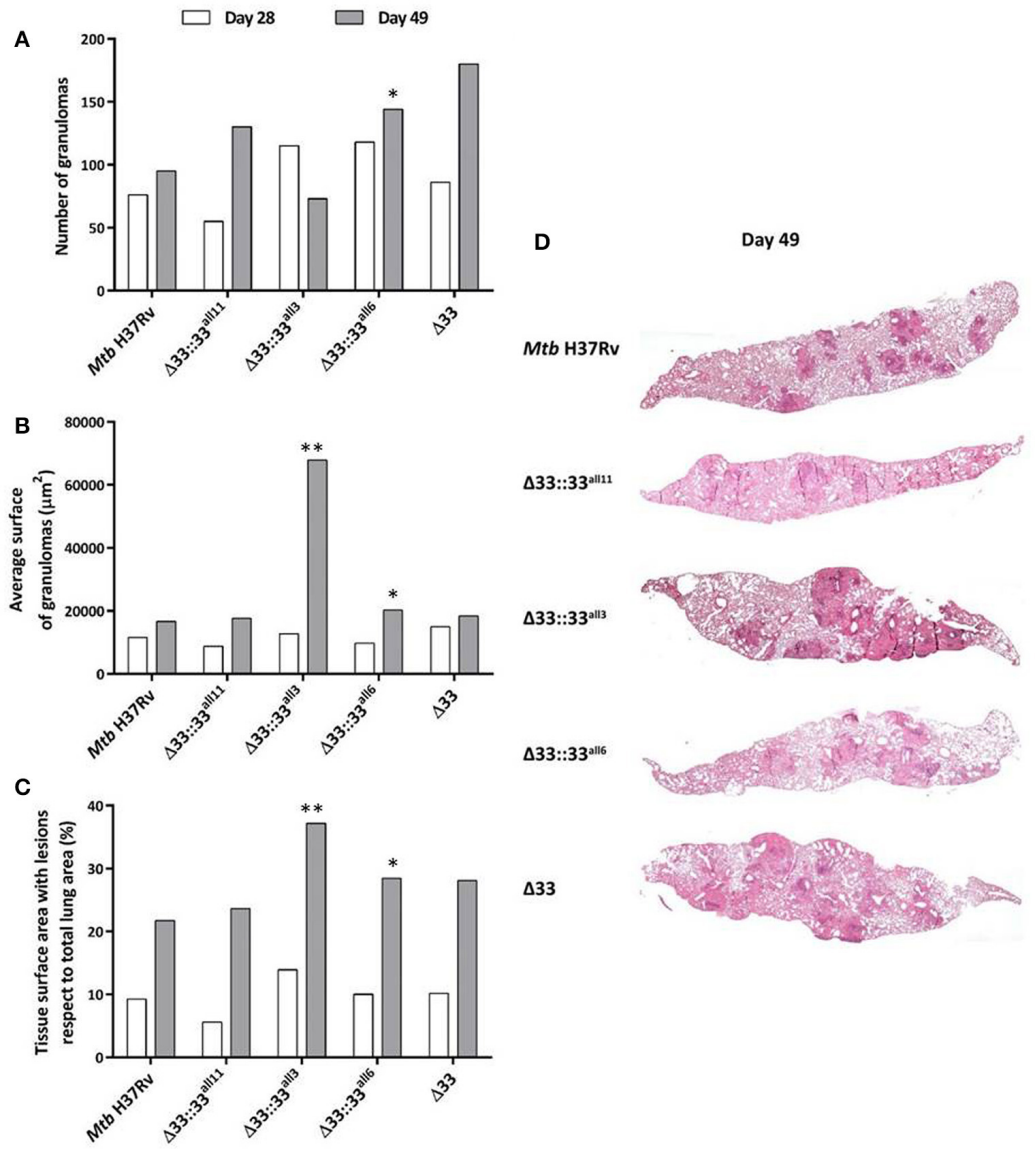
**The lack and truncation of PE_PGRS33 is responsible for an increased virulence phenotype of *Mtb* in lung tissue during the chronic/persistent *Mtb* infection in mice**. Quantitative and qualitative histopathological analysis were performed on lung sections of mice infected with *Mtb* H37Rv, the *Mtb*Δ33 mutant and complemented strains (*Mtb*Δ33::33^all11^, *Mtb*Δ33::33^all3^, and *Mtb*Δ33::33^all6^). At least six lung sections *per* mouse for all 5 groups were analyzed in different points. Extent of tissue damage was assessed as: **(A)** number of granulomas; **(B)** average surface of granulomas; and **(C)** percentage of tissue surface area with lesions with respect to total lung area. **(D)** Granuloma extension and distribution in lung tissue were evaluated after performing histopathological analysis of lung sections stained with H&E at days 49 post-infection (200x magnification). Representative slides are shown. ^*^p < 0.05 and ^**^p < 0.01 (Student's *t*-test).

For all 5 strains tested in *in vivo* infections (*Mtb* H37Rv, *Mtb*Δ33::33^all11^, *Mtb*Δ33::33^all3^, *Mtb*Δ33::33^all6^, and *Mtb*Δ33), both quantitative and qualitative histopathological examinations were carried out on lung sections of mice sacrificed at day 28 and day 49 post-infection (Figure 8). At day 28 post-infection, no major differences were observed between the *Mtb* strains tested, in terms of number and extension of granulomas and net pulmonary area with lesions (Figures 8A–C). Similarly, at day 49 post-infection, apart from *Mtb*Δ33::33^all3^ (*p* < 0.01) and to a lesser extent *Mtb*Δ33::33^all6^, no significant differences were found between all other strains analyzed (Figures 8A–D). However, a higher, but not significant number of granulomas was enumerated for *Mtb*Δ33::33^all11^, *Mtb*Δ33::33^all6^, and *Mtb*Δ33, in particular, compared to the parental strain (Figure 8A). Conversely, the lungs of mice infected with *Mtb*Δ33::33^all3^ were characterized by a reduction in the number of granulomas compared with those of mice infected with *Mtb* H37Rv (*p* < 0.01; Figure 8A). Surprisingly, based on the size of granulomas and net pulmonary area with lesions, *Mtb*Δ33::33^all3^ caused more extensive tissue damage (*p* < 0.01) compared to the parental strain, with major pulmonary confluent lesions which justify the low number of granulomas detected in the lungs (Figures 8B,D). At both day 28 and day 49 post-infection, the distribution and percentage of cells with acid-fast bacilli was similar for all analyzed strains, apart from *Mtb*Δ33::33^all3^, which was associated with a higher number of AFB compared to the parental strain at day 49 post-infection. This last observation was consistent with the features and extent of histopathological lesions shown in Supplementary Figures 5, 6. Taken together, these results suggest an interesting increased capacity of *Mtb* to evoke lung tissue damage in murine model, when the PE_PGRS33 protein is truncated or completely absent.

### The lack and truncation of PE_PGRS33 is responsible for the extracellular localization of *Mtb* in murine lung tissue during the chronic persistent TB disease stages

**Figure 9:**
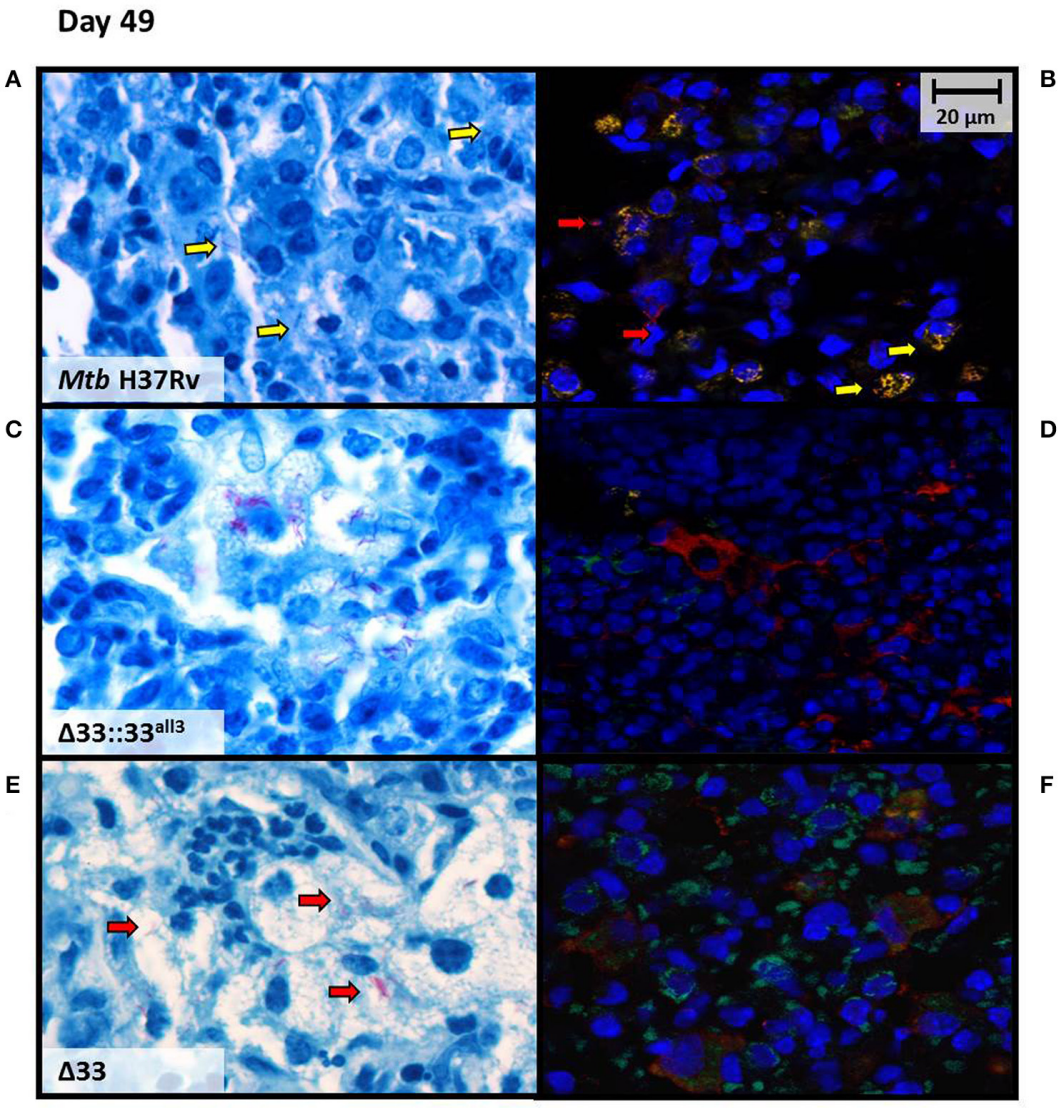
***Mtb*** **lacking PE_PGRS33 or expressing the truncated allele 3 mainly localizes extracellularly in murine lungs during the chronic/persistent TB stages**. Cellular localization of bacilli was investigated at day 49 post-infection on lung sections of mice infected with the *Mtb* H37Rv **(A,B)**, *Mtb*Δ33::33^all3^ **(C,D)** and *Mtb*Δ33 **(E,F)** strains, by performing immunofluorescence assay **(B,D,F)** and comparing results with those obtained from observation of ZN-stained lung sections **(A,C,E)**. Immunofluorescence was carried out by using anti-F4/80 and anti-MT 16 kDa antigen, as markers of macrophages (green) and bacilli (red), respectively. Colocalization of macrophages and bacteria resulted in yellow signals. Representative images are shown.

At higher magnification, lung sections from mice infected with *Mtb*Δ33, *Mtb*Δ33::33^all3^, and *Mtb*Δ33::33^all6^ were remarkably characterized by a high number of foamy macrophages at day 49 post-infection compared with the sections obtained from the other groups of mice (Supplementary Figure 6). Moreover, AFB positive cells remained stable in *Mtb* H37Rv, *Mtb*Δ33::33^all11^, *Mtb*Δ33::33^all6^, and *Mtb*Δ33 strains, but *Mtb*Δ33::33^all3^ showed a higher number of lesions, a reduced percentage of positive cells and a high number of AFB found extracellularly (Supplementary Figure 6). To better characterize bacilli localization of *Mtb*Δ33::33^all3^ compared to *Mtb* H37Rv and *Mtb*Δ33 in infected tissues, immunofluorescence was performed by using two markers, anti-F4/80 and anti-MT 16 kDa antigen, to point out macrophages and bacilli, respectively. The intra and extracellular position of bacilli was then evaluated by comparing the results of immune-colocalization with those obtained from observation of Ziehl-Neelsen (ZN)-stained lung sections (Figure 9). As shown in Figure 9B, a yellow signal was repeatedly observed in lung sections of mice infected with *Mtb* H37Rv and derived from overlapping between red and green signals, which label mycobacteria and macrophages, respectively. An evident separation between the red and green fluorescence of *Mtb*Δ33::33^all3^ and macrophages, respectively, revealed the predominant extracellular position of this strain (Figure 9D). Also for *Mtb*Δ33, it was demonstrated that the two fluorescence signals were detected within the same area although did not overlap regularly (Figure 9F). Moreover, further differences, in terms of immune cells composition, were also observed in lung tissue sections, as showed in ZN images (Figures 9A,C,E). Particularly, a prevalence of macrophages and some lymphocyte were found in lung tissue of mice infected with *Mtb* H37Rv (Figure 9A). Similarly, lungs of mice infected with both *Mtb*Δ33::33^all3^ and *Mtb*Δ33 were characterized by a predominance of foamy macrophages, although the former also contained apoptotic cells and the latter polymorphonuclear cells (Figures 9C,E). These further investigations allowed to point out a mainly extracellular localization for the *Mtb*Δ33::33^all3^ and *Mtb*Δ33 strains, showing how the lack or truncation of PE_PGRS33 may affect the cellular localization of *Mtb* during the chronic persistent TB disease stages.

## Discussion

Members of the MTBC show high homogeneity at genomic level (99.9%), with most of the genetic variability resting on two gene families, PE and PPE (Brennan and Delogu, 2002; McEvoy et al., 2012; Copin et al., 2014; Fishbein et al., 2015). Several genes of the PE_PGRS subfamily show high genetic variability, including the *pe_pgrs33* gene which was shown to account for a wide heterogeneity of polymorphisms mainly occurring in the PGRS domain (Talarico et al., 2005, 2007). Sequence variations in this gene have been correlated with clinical and epidemiological TB phenotypes (Talarico et al., 2007; Wang et al., 2011), supporting the hypothesis that surface exposed PE_PGRS proteins may be involved in antigenic variability (Cole et al., 1998; Banu et al., 2002; Delogu et al., 2004). However, despite several studies recognized the immunomodulatory role of PE_PGRS33 (Dheenadhayalan et al., 2006; Balaji et al., 2007; Basu et al., 2007), it remains still unclear whether and how *pe_pgrs33* polymorphisms may affect the pathogenesis and virulence of *Mtb*. To address this issue, we investigated the genetic variability of *pe_pgrs33* in a collection of 135 randomly selected MTBC clinical isolates and assessed in *in vitro* and *in vivo* models the impact of large sequence deletions on PE_PGRS33 function at early and late phases of *Mtb* infection.

In our collection of 135 genotyped MTBC clinical isolates, we identified 19 *pe_pgrs33* alleles: 12 corresponded to alleles already characterized in previous studies, including *pe_pgrs33* of *Mtb* H37Rv, while the other 7 alleles were newly identified in this study. As shown in Figure 1, where each MTBC clinical strain was color-coded by the respective *pe_pgrs33* allele, the overall association observed between specific alleles and MTBC superlineages or lineages suggests a clustering of *pe_pgrs33* alleles during the evolution of *Mtb*. Moreover, we obtained a dN/dS ratio of 0.64, which being below 1 indicates that *pe_pgrs33* is under a purifying selection. Contrary to the possible involvement of PE_PGRS33 in the antigenic variability of *Mtb* and in agreement with a recent work of Copin et al. (2014), our results support the evidence that *pe_pgrs33* is under a biologic pressure to prevent polymorphisms, which may impair the key functional role of this protein in the biology of *Mtb*. Previous studies correlated naturally-occurring polymorphisms in *pe_pgrs33* with TB clinical features: genetic variations in this gene have been associated with TB meningitis in children (Wang et al., 2011) and large in-frame indels and frameshift mutations have been correlated with the absence of cavitation in the lungs (Talarico et al., 2007). In these circumstances, it appears that large polymorphisms in *pe_pgrs33* do not affect the virulence of *Mtb*, since TB in children and extrapulmonary TB may present with severe clinical patterns, which, however, do not warrant an efficient transmission of *Mtb*. Moreover, it is interesting to note that the *pe_pgrs33* gene is absent in the genome of *M. marinum*, which contains more than 100 *pe_pgrs* genes (Delogu et al., 2008), and in smooth tubercle bacilli (STB) (Supply et al., 2013), suggesting that *pe_pgrs33* belongs to the panel of genes that could have been acquired by MTBC to gain additional virulence and persistence mechanisms (Supply et al., 2013). Together, all of these evidences suggest the potential critical role of *pe_pgrs33* for the successful transmission of *Mtb* in humans.

We recently characterized *in vitro* the *Mtb* mutant for *pe_pgrs33* (*Mtb*Δ33), demonstrating that the lack of PE_PGRS33 results in a significant impairment of the *Mtb* entry capacity into macrophages (Palucci et al., 2016), and proposed that the binding of PE_PGRS33 to TLR2 may reasonably activate the inside-out signaling, which contributes to the entry of *Mtb* into macrophages (Palucci et al., 2016). In this study, when the *Mtb*Δ33 mutant strain was complemented with plasmids encoding selected *pe_pgrs33* alleles and these *Mtb* strains were used to infect murine macrophages, we observed complementation of the parental strain phenotype with *pe_pgrs33* alleles showing both small genetic variations and large in-frame deletions. Conversely, the truncated PE_PGRS33 protein encoded by 33^all3^, which contained a frameshift mutation as major variation, was responsible for an impaired ability of *Mtb*Δ33::33^all3^ to enter murine macrophages, similar to that one of the *Mtb*Δ33 mutant (Palucci et al., 2016). In infection of human macrophages, the *Mtb* strains expressing *pe_pgrs33* alleles with large deletions did not fully restore the parental strain phenotype and the defect in *Mtb* entry was again particularly marked for *Mtb*Δ33::33^all3^. Moreover, the replication capacity of the *Mtb*Δ33 and complemented strains during infection of human macrophages and PBMCs was not affected by mutations in *pe_pgrs33* and the level of cytokines secreted by the innate immunity were not perturbed at 72 h post-infection. These results suggest that the absence of PE_PGRS33 or the presence of mutations, which occur in the gene region encoding the PGRS domain and determine an extensive and dramatic change in the protein sequence and presumably structure, do not impact the ability of *Mtb* to survive and multiply into macrophages. Potential differences in the ability of PE_PGRS33 to interact with cell surface receptors of murine and human macrophages, probably due to the polymorphic nature of the variable region of TLR2 (Grabiec et al., 2004), could explain the differences observed between the two cell models used for infections, although our hypothesis would require further investigations.

The role of PE_PGRS33 in the pathogenesis and virulence of *Mtb* was also investigated *in vivo* by infecting mice *via* the intranasal route and assessing the impact of major variations (frameshift and in-frame deletions) occurring in *pe_pgrs33*, in terms of bacterial burden and extent of tissue damage in murine lungs at different time points. Compared to the parental strain, no differences were observed in the ability of the *Mtb*Δ33 mutant and complemented strains to colonize the lung tissue of mice at day 0 post-infection, suggesting that the defect in the macrophage cell entry phenotype observed *in vitro* particularly for the *Mtb*Δ33::33^all3^ and *Mtb*Δ33 mutant strains does not translate in an enhanced killing *in vivo*, during the early stages of the infectious process. Surprisingly, while the replication capacity of *Mtb*Δ33 was comparable with those of *Mtb* H37Rv at day 28 post-infection, a significant higher bacterial burden in the lung tissue of mice was observed in absence of PE_PGRS33 and was accompanied by more extensive histopathological lesions in the *Mtb*Δ33 mutant compared to the parental strain at day 49 post-infection. These results point toward a key role of PE_PGRS33 in the immunopathogenesis of TB, specifically during the chronic/persistent steps of the infectious process in mice.

Interestingly, quantitative and qualitative histopathology analysis of lung sections of mice infected with the *Mtb*Δ33::33^all3^ revealed a significant extension of lung tissue damage and lesions even much more pronounced compared with those observed in the lung tissue of mice infected with the *Mtb*Δ33 mutant strain. These results suggest that major variations, such as the frameshift deletion expressed by the *Mtb*Δ33::33^all3^, are associated with a pattern of lung tissue damage and virulence phenotype similar, if not more remarkable, to that observed with *Mtb*Δ33 during the chronic stages of the infectious process. These results further highlight the impact that subtle changes in the expression of PE_PGRS33 in host tissues may have on the pathogenesis of TB.

In most cases, genetic deletions of highly conserved genes negatively affect the bacterial virulence resulting in an attenuated phenotype. However, a number of studies provided evidences of an hypervirulent phenotype following infection with *Mtb* mutant strains, wherein individual genes of different functional classes and crucial for the bacilli have been disrupted (ten Bokum et al., 2008). In this study, we observed that the disruption or truncation of *pe_pgrs33*, a gene important in the pathogenesis of *Mtb*, resulted in a phenotype with enhanced virulence of the *Mtb*Δ33 mutant and *Mtb*Δ33::33^all3^ strains in the lungs of mice at day 49 post infection. These results suggest a role of PE_PGRS33 during the chronic/persistent phase of the disease, similarly to what observed for PE_PGRS30 (Iantomasi et al., 2012) and other PE_PGRSs (Kruh et al., 2010). These unexpected and only apparently controversial results, when compared with what observed in macrophages *in vitro*, deserve further investigation in animal models known to better mimic human TB (Orme and Basaraba, 2014). However, regarding the enhanced virulence observed for the *Mtb*Δ33::33^all3^ strain, we do not think that the frameshift mutation in 33^all3^ results in a loss of function for the entire PE_PGRS33 protein, but rather we hypothesize that the frameshift allele, which we identified exclusively among all *Mtb* strains of our collection belonging to the ancient superlineage 1 or EAI, could represent the real ancestral gene. In this perspective and from an evolutionary point of view, allele variants of *pe_pgrs33*, including the gene of *Mtb* H37Rv, may have evolved from the frameshift allele to trigger a yet unidentified pattern of immunomodulation at local level in the lung lesions that in human may be instrumental to promote tissue damage and ultimately to the successful transmission of *Mtb* to new hosts (Comas and Gagneux, 2011). Based on previous evidences about the diversity of selective pressures acting on *pe_pgrs* genes (Copin et al., 2014), in future studies, it would be interesting to deepen the impact of *pe_pgrs33* alleles during the infectious process of *Mtb*, in the context of the respective native strains.

PE_PGRS33 with its different polymorphisms, and lack of PE_PGRS33 thereof, can affect the immunomodulatory properties of *Mtb* in host tissues in at least two ways, particularly during the chronic/persistent steps of *Mtb* infection when bacilli loads are significant. First, direct interaction of PE_PGRS33 on *Mtb* surface with TLR2 may impact the cytokine milieu within the lesions and host cells viability (Dheenadhayalan et al., 2006; Balaji et al., 2007; Basu et al., 2007; Zumbo et al., 2013), which may clearly affect the inflammatory environment in the lesions. Second, the defect of *Mtb*Δ33 to enter in host macrophages (Palucci et al., 2016) may result in a higher number of extracellular bacilli, which are known to differentially modulate host immune responses at the site of infection (Orme, 2014). In this context, the phenotype observed *in vivo* for the *Mtb*Δ33 mutant and complemented strains with 33^all11^, 33^all3^, and 33^all6^ point for a key role of this protein in the immunopathogenesis of TB. However, given the results obtained in mice, characterization of the immunomodulatory mechanisms responsible for the role of PE_PGRS33 in TB pathogenesis shall preferentially be carried on relevant human model of TB (Orme and Basaraba, 2014). Hence, while it remains to be elucidated how PE_PGRS33 affect *Mtb* virulence in humans, the results of this study indicate that the immunomodulatory properties of PE_PGRS33 impact on the immunopathogenesis of TB.

## Author contributions

SC, IP, MSali, and GD designed the study; DG, FR, MSanguinetti, RM, SR, PB, and GD contributed reagents, materials and analysis tools; SC, IP, RI, TC, MM, FD, SJ, and EP performed the experiments; all authors analyzed the data and interpreted the results; SC and GD wrote the manuscript. All authors reviewed and discussed the manuscript.

## Funding

This work was supported by a grant from the Ministry of Health of Italy “Ricerca Finalizzata” RF-2011-02348713 awarded to GD and, for the animal experiments, financial support was provided by the European Community (ERC-STG INTRACELLTB Grant n° 260901).

### Conflict of interest statement

The authors declare that the research was conducted in the absence of any commercial or financial relationships that could be construed as a potential conflict of interest.

## References

[B1] Allix-Béguec C. Harmsen D. Weniger T. Supply P. Niemann S. (2008). Evaluation and strategy for use of MIRU-VNTRplus, a multifunctional database for online analysis of genotyping data and phylogenetic identification of *Mycobacterium tuberculosis* complex isolates. J. Clin. Microbiol. 46, 2692–2699. 10.1128/JCM.00540-08 18550737 PMC2519508

[B2] Balaji K. N. Goyal G. Narayana Y. Srinivas M. Chaturvedi R. Mohammad S. (2007). Apoptosis triggered by Rv1818c, a PE family gene from *Mycobacterium tuberculosis* is regulated by mitochondrial intermediates in T cells. Microbes Infect. 9, 271–281. 10.1016/j.micinf.2006.11.013 17223373

[B3] Banu S. Honoré N. Saint-Joanis B. Philpott D. Prévost M. C. Cole S. T. (2002). Are the PE-PGRS proteins of *Mycobacterium tuberculosis* variable surface antigens? Mol. Microbiol. 44, 9–19. 10.1046/j.1365-2958.2002.02813.x 11967065

[B4] Basu S. Pathak S. K. Banerjee A. Pathak S. Bhattacharyya A. Yang Z.. (2007). Execution of macrophage apoptosis by PE_PGRS33 of *Mycobacterium tuberculosis* is mediated by Toll-like receptor 2-dependent release of tumor necrosis factor-alpha. J. Biol. Chem. 282, 1039–1050. 10.1074/jbc.M604379200 17095513

[B5] Brennan M. J. Delogu G. (2002). The PE multigene family: a ‘molecular mantra’ for mycobacteria. Trends Microbiol. 10, 246–249. 10.1016/S0966-842X(02)02335-1 11973159

[B6] Brennan M. J. Delogu G. Chen Y. Bardarov S. Kriakov J. Alavi M.. (2001). Evidence that mycobacterial PE_PGRS proteins are cell surface constituents that influence interactions with other cells. Infect. Immun. 69, 7326–7333. 10.1128/IAI.69.12.7326-7333.2001 11705904 PMC98818

[B7] Cascioferro A. Daleke M. H. Ventura M. Donà V. Delogu G. Palù G.. (2011). Functional dissection of the PE domain responsible for translocation of PE_PGRS33 across the mycobacterial cell wall. PLoS ONE 6:e27713. 10.1371/journal.pone.0027713 22110736 PMC3218021

[B8] Cole S. T. Brosch R. Parkhill J. Garnier T. Churcher C. Harris D.. (1998). Deciphering the biology of *Mycobacterium tuberculosis* from the complete genome sequence [see comments] [published erratum appears in Nature 1998 Nov 12;396(6707):190]. Nature 393, 537–544. 10.1038/31159 9634230

[B9] Comas I. Coscolla M. Luo T. Borrell S. Holt K. E. Kato-Maeda M.. (2013). Out-of-Africa migration and Neolithic coexpansion of *Mycobacterium tuberculosis* with modern humans. Nat. Genet. 45, 1176–1182. 10.1038/ng.2744 23995134 PMC3800747

[B10] Comas I. Gagneux S. (2011). A role for systems epidemiology in tuberculosis research. Trends Microbiol. 19, 492–500. 10.1016/j.tim.2011.07.002 21831640 PMC3184389

[B11] Copin R. Coscolla M. Seiffert S. N. Bothamley G. Sutherland J. Mbayo G.. (2014). Sequence diversity in the pe_pgrs genes of *Mycobacterium tuberculosis* is independent of human T cell recognition. MBio 5, e00960–e00913. 10.1128/mbio.00960-13 24425732 PMC3903279

[B12] Delogu G. Cole S. T. Brosch R. (2008). The PE and PPE Protein Families of *Mycobacterium tuberculosis*, in Handbook of Tuberculosis. eds Kaufmann S. H. Rubin E.(Weinheim: Wiley-VCH Verlag GmbH & Co. KGaA), 131–150.

[B13] Delogu G. Pusceddu C. Bua A. Fadda G. Brennan M. J. Zanetti S. (2004). Rv1818c-encoded PE_PGRS protein of *Mycobacterium tuberculosis* is surface exposed and influences bacterial cell structure. Mol. Microbiol. 52, 725–733. 10.1111/j.1365-2958.2004.04007.x 15101979

[B14] De Maio F. Maulucci G. Minerva M. Anoosheh S. Palucci I. Iantomasi R.. (2014). Impact of protein domains on PE_PGRS30 polar localization in Mycobacteria. PLoS ONE 9:e112482. 10.1371/journal.pone.0112482 25390359 PMC4229189

[B15] Dheenadhayalan V. Delogu G. Brennan M. J. (2006). Expression of the PE_PGRS 33 protein in Mycobacterium smegmatis triggers necrosis in macrophages and enhanced mycobacterial survival. Microbes Infect. 8, 262–272. 10.1016/j.micinf.2005.06.021 16203168

[B16] Firdessa R. Berg S. Hailu E. Schelling E. Gumi B. Erenso G.. (2013). Mycobacterial lineages causing pulmonary and extrapulmonary tuberculosis, Ethiopia. Emer. Infect. Dis. 19, 460–463. 10.3201/eid1903.120256 23622814 PMC3647644

[B17] Fishbein S. van Wyk N. Warren R. M. Sampson S. L. (2015). Phylogeny to function: PE/PPE protein evolution and impact on *Mycobacterium tuberculosis* pathogenicity. Mol. Microbiol. 96, 901–916. 10.1111/mmi.12981 25727695

[B18] Gagneux S. (2012). Host-pathogen coevolution in human tuberculosis. Philos. Trans. R. Soc. Lond. B. Biol. Sci. 367, 850–859. 10.1098/rstb.2011.0316 22312052 PMC3267123

[B19] Gagneux S. Small P. M. (2007). Global phylogeography of *Mycobacterium tuberculosis* and implications for tuberculosis product development. Lancet Infect. Dis. 7, 328–337. 10.1016/S1473-3099(07)70108-1 17448936

[B20] Gey van Pittius N. C. Sampson S. L. Lee H. Kim Y. van Helden P. D. Warren R. M. (2006). Evolution and expansion of the *Mycobacterium tuberculosis* PE and PPE multigene families and their association with the duplication of the ESAT-6 (esx) gene cluster regions. BMC Evol. Biol. 6:95. 10.1186/1471-2148-6-95 17105670 PMC1660551

[B21] Grabiec A. Meng G. Fichte S. Bessler W. Wagner H. Kirschning C. J. (2004). Human but not murine toll-like receptor 2 discriminates between tri-palmitoylated and tri-lauroylated peptides. J. Biol. Chem. 279, 48004–48012. 10.1074/jbc.M405311200 15342637

[B22] Gutierrez M. C. Brisse S. Brosch R. Fabre M. Omaïs B. Marmiesse M.. (2005). Ancient origin and gene mosaicism of the progenitor of *Mycobacterium tuberculosis*. PLoS Pathog. 1:e5. 10.1371/journal.ppat.0010005 16201017 PMC1238740

[B23] Hershberg R. Lipatov M. Small P. M. Sheffer H. Niemann S. Homolka S.. (2008). High functional diversity in *Mycobacterium tuberculosis* driven by genetic drift and human demography. PLoS Biol. 6:e311. 10.1371/journal.pbio.0060311 19090620 PMC2602723

[B24] Iantomasi R. Sali M. Cascioferro A. Palucci I. Zumbo A. Soldini S.. (2012). PE_PGRS30 is required for the full virulence of *Mycobacterium tuberculosis*. Cell. Microbiol. 14, 356–367. 10.1111/j.1462-5822.2011.01721.x 22050772

[B25] Kamerbeek J. Schouls L. Kolk A. van A. M. van S. D. Kuijper S.. (1997). Simultaneous detection and strain differentiation of *Mycobacterium tuberculosis* for diagnosis and epidemiology. J. Clin. Microbiol. 35, 907–914. 9157152 10.1128/jcm.35.4.907-914.1997 PMC229700

[B26] Kruh N. A. Troudt J. Izzo A. Prenni J. Dobos K. M. (2010). Portrait of a pathogen: the *Mycobacterium tuberculosis* proteome *in vivo*. PLoS ONE 5:e13938. 10.1371/journal.pone.0013938 21085642 PMC2978697

[B27] McEvoy C. R. Cloete R. Müller B. Schurch A. C. van Helden P. D. Gagneux S.. (2012). Comparative analysis of *Mycobacterium tuberculosis* pe and ppe genes reveals high sequence variation and an apparent absence of selective constraints. PLoS ONE 7:e30593. 10.1371/journal.pone.0030593 22496726 PMC3319526

[B28] Mukhopadhyay S. Balaji K. N. (2011). The PE and PPE proteins of *Mycobacterium tuberculosis*. Tuberculosis (Edinb). 91, 441–447. 10.1016/j.tube.2011.04.004 21527209

[B29] Orme I. M. (2014). A new unifying theory of the pathogenesis of tuberculosis. Tuberculosis (Edinb). 94, 8–14. 10.1016/j.tube.2013.07.004 24157189 PMC3877201

[B30] Orme I. M. Basaraba R. J. (2014). The formation of the granuloma in tuberculosis infection. Semin. Immunol. 26, 601–609. 10.1016/j.smim.2014.09.009 25453231

[B31] Palucci I. Camassa S. Cascioferro A. Sali M. Anoosheh S. Zumbo A.. (2016). PE_PGRS33 contributes to *Mycobacterium tuberculosis* Entry in Macrophages through Interaction with TLR2. PLoS ONE 11:e0150800. 10.1371/journal.pone.0150800 26978522 PMC4792380

[B32] Portevin D. Gagneux S. Comas I. Young D. (2011). Human macrophage responses to clinical isolates from the *Mycobacterium tuberculosis* complex discriminate between ancient and modern lineages. PLoS Pathog. 7:e1001307. 10.1371/journal.ppat.1001307 21408618 PMC3048359

[B33] Poulet S. Cole S. T. (1995). Characterization of the highly abundant polymorphic GC-rich-repetitive sequence (PGRS) present in *Mycobacterium tuberculosis*. Arch. Microbiol. 163, 87–95. 10.1007/BF00381781 7710330

[B34] Rozas J. Sánchez-DelBarrio J. C. Messeguer X. Rozas R. (2003). DnaSP, DNA polymorphism analyses by the coalescent and other methods. Bioinformatics 19, 2496–2497. 10.1093/bioinformatics/btg359 14668244

[B35] Sampson S. L. (2011). Mycobacterial PE/PPE proteins at the host-pathogen interface. Clin. Dev. Immunol. 2011:497203. 10.1155/2011/497203 21318182 PMC3034920

[B36] Soldini S. Palucci I. Zumbo A. Sali M. Ria F. Manganelli R.. (2011). PPE_MPTR genes are differentially expressed by *Mycobacterium tuberculosis in vivo*. Tuberculosis (Edinb). 91, 563–568. 10.1016/j.tube.2011.08.002 21890414

[B37] Supply P. Marceau M. Mangenot S. Roche D. Rouanet C. Khanna V.. (2013). Genomic analysis of smooth tubercle bacilli provides insights into ancestry and pathoadaptation of *Mycobacterium tuberculosis*. Nat. Genet. 45, 172–179. 10.1038/ng.2517 23291586 PMC3856870

[B38] Talarico S. Cave M. D. Foxman B. Marrs C. F. Zhang L. Bates J. H.. (2007). Association of *Mycobacterium tuberculosis* PE PGRS33 polymorphism with clinical and epidemiological characteristics. Tuberculosis (Edinb). 87, 338–346. 10.1016/j.tube.2007.03.003 17475562 PMC2093954

[B39] Talarico S. Cave M. D. Marrs C. F. Foxman B. Zhang L. Yang Z. (2005). Variation of the *Mycobacterium tuberculosis* PE_PGRS 33 gene among clinical isolates. J. Clin. Microbiol. 43, 4954–4960. 10.1128/JCM.43.10.4954-4960.2005 16207947 PMC1248487

[B40] Tamura K. Stecher G. Peterson D. Filipski A. Kumar S. (2013). MEGA6: molecular evolutionary genetics analysis version 6.0. Mol. Biol. Evol. 30, 2725–2729. 10.1093/molbev/mst197 24132122 PMC3840312

[B41] ten Bokum A. M. Movahedzadeh F. Frita R. Bancroft G. J. Stoker N. G. (2008). The case for hypervirulence through gene deletion in *Mycobacterium tuberculosis*. Trends Microbiol. 16, 436–441. 10.1016/j.tim.2008.06.003 18701293

[B42] van Embden J. D. Cave M. D. Crawford J. T. Dale J. W. Eisenach K. D. Gicquel B.. (1993). Strain identification of *Mycobacterium tuberculosis* by DNA fingerprinting: recommendations for a standardized methodology. J. Clin. Microbiol. 31, 406–409. 8381814 10.1128/jcm.31.2.406-409.1993 PMC262774

[B43] Wang J. Huang Y. Zhang A. Zhu C. Yang Z. Xu H. (2011). DNA polymorphism of *Mycobacterium tuberculosis* PE_PGRS33 gene among clinical isolates of pediatric TB patients and its associations with clinical presentation. Tuberculosis (Edinb). 91, 287–292. 10.1016/j.tube.2011.05.001 21664871

[B44] Wirth T. Hildebrand F. Allix-Béguec C. Wölbeling F. Kubica T. Kremer K.. (2008). Origin, spread and demography of the *Mycobacterium tuberculosis* complex. PLoS Pathog. 4:e1000160. 10.1371/journal.ppat.1000160 18802459 PMC2528947

[B45] World Health Organization (2016). Global Tuberculosis Report 2016. Geneva.

[B46] Zumbo A. Palucci I. Cascioferro A. Sali M. Ventura M. D'Alfonso P.. (2013). Functional dissection of protein domains involved in the immunomodulatory properties of PE_PGRS33 of *Mycobacterium tuberculosis*. Pathog. Dis. 69, 232–239. 10.1111/2049-632X.12096 24106104

