# Supplementary material for: Impact of pe_pgrs33 Gene Polymorphisms on Mycobacterium tuberculosis Infection and Pathogenesis
Source: Front Cell Infect Microbiol. 2017 Apr 21;7:137. doi: 10.3389/fcimb.2017.00137 (PMC5399086; doi:10.3389/fcimb.2017.00137)
Supplement: Supplementary file 1 [file Table1.PDF]

**Supplementary Table 1. Details of the primers used in this study.**

| Primer | 5'→3' | Note |
|---|---|---|
| **PE-PGRS33F1** | GCCGACAAGGCGTCGTGAGA | Primer pair used to amplify and sequence all *pe_pgrs33* alleles (Wang et al., 2011) |
| **PE-PGRS33R1** | CCGCCGAAGTGTAAGCCGGG | |
| **PGRS0778F** | CACCAATACCGCCCACCCCACCAC | Primers used to sequence and confirm all polymorphisms identified in the *pe_pgsr33* alleles (Talarico et al., 2005) |
| **PGRS0778R** | GTGGTGGGGTGGGCGGTATTGGTG | |
| **PGRS0660R** | CGGCGGAGACGGCGGGTTGTT | |
| **PG335Hn-338bp** | AC<u>AAGCTT</u>TTCCTCGACGAAAGTACCTCGGCG HindIII | Primers used to amplify 5 *pe_pgrs33* alleles (under the control of their native promoter) subsequently cloned in pMV306 vector (Palucci et al., 2016) |
| **18c3AXb** | AC<u>TCTAGA</u>CGGTAACCCGTTCATCCCGTTCTT XbaI | |
| **PMV306 MF** | GTTATCCCCTGATTCTGTGGA | Primer pair which specifically anneals to the multicloning site of pMV306 vector 93bp upstream XbaI site and 109bp downstream HindIII site, respectively |
| **PMV306 MR** | ATCACCGCGGCCATGATG | |
