# Supplementary material for: Impact of pe_pgrs33 Gene Polymorphisms on Mycobacterium tuberculosis Infection and Pathogenesis
Source: Front Cell Infect Microbiol. 2017 Apr 21;7:137. doi: 10.3389/fcimb.2017.00137 (PMC5399086; doi:10.3389/fcimb.2017.00137)
Supplement: Supplementary file 2 [file Table2.PDF]

Supplementary Table 2. Details of the 19 *pe_pgrs33* alleles identified in this study.

pe_pgrs33 allele	Genetic variation	Position (bp)	Amino acid variation	Position (aa)	Protein domain
1	sSNP	207	none	69	PE
	nsSNP	347	Pro → Leu	116	TM
	– 72bp	416-487	–4 Gly Gly X[a]	140-163	PGRS
	sSNP	717	none	239	PGRS
	– 1bp	1014	–28 Gly Gly X[a]	338-498	PGRS
	(+ 9bp)	(1240)	(+ 1 Gly Gly Ala)	(413)	(PGRS)
2	sSNP	717	none	239	PGRS
	– 1bp	1014	–28 Gly Gly X[a]	338-498	PGRS
	(+ 9bp)	(1240)	(+ 1 Gly Gly Ala)	(413)	(PGRS)
3	sSNP	582	none	194	PGRS
	sSNP	717	none	239	PGRS
	– 1bp	1014	–28 Gly Gly X[a]	338-498	PGRS
	(+ 9bp)	(1240)	(+ 1 Gly Gly Ala)	(413)	(PGRS)
4	sSNP	717	none	239	PGRS
	+ 9bp	1240	+ 1 Gly Gly Ala	413	PGRS
5	nsSNP	697	Gly → Ser	233	PGRS
	sSNP	717	none	239	PGRS
	+ 9bp	1240	+ 1 Gly Gly Ala	413	PGRS
6	– 72bp	416-487	–4 Gly Gly X[a]	140-163	PGRS
	sSNP	717	none	239	PGRS
	+ 9bp	1240	+ 1 Gly Gly Ala	413	PGRS
7	nsSNP	278	Ala → Asp	93	TM
	– 9bp	640-648	–1 Gly Gly Ala	214-216	PGRS
8	nsSNP	665	Gly → Asp	222	PGRS
9	– 9bp	1129-1137	–1 Gly Gly Ala	377-379	PGRS

**10**	nsSNP	820	Gly → Ser	274	PGRS
**11**	none[b]				
**12**	+ 18bp	597	+ 2 Gly Gly Ala	199	PGRS
	sSNP	717	none	239	PGRS
	+ 9bp	1240	+ 1 Gly Gly Ala	413	PGRS
**13**	– 9bp	640-648	–1 Gly Gly Ala	214-216	PGRS
**14**	nsSNP	493,494	Asn → Ala	165	PGRS
	sSNP	717	none	239	PGRS
	+ 9bp	1240	+ 1 Gly Gly Ala	413	PGRS
**15**	nsSNP	529	Ser → Ala	177	PGRS
**16**	sSNP	717	none	239	PGRS
	sSNP	1092	none	364	PGRS
	+ 9bp	1240	+ 1 Gly Gly Ala	413	PGRS
**17**	– 39bp	547-585	–3 Gly Gly Ala	183-195	PGRS
	sSNP	717	none	239	PGRS
	+ 9bp	1240	+ 1 Gly Gly Ala	413	PGRS
**18**	sSNP	717	none	239	PGRS
	– 42bp	772-813	–3 Gly Gly X[a]	257-270	PGRS
	+ 9bp	1240	+ 1 Gly Gly Ala	413	PGRS
**19**	nsSNP	235	Ala → Thr	79	PE
	sSNP	717	none	239	PGRS
	– 42bp	772-813	–3 Gly Gly X[a]	257-270	PGRS

[a] X any amino acid.

[b] none compared to *pe_pgrs33* gene of *Mtb* H37Rv (NC_000962.3).
