# Supplementary material for: Impact of pe_pgrs33 Gene Polymorphisms on Mycobacterium tuberculosis Infection and Pathogenesis
Source: Front Cell Infect Microbiol. 2017 Apr 21;7:137. doi: 10.3389/fcimb.2017.00137 (PMC5399086; doi:10.3389/fcimb.2017.00137)
Supplement: Supplementary file 3 [file Presentation1.PDF]

Supplementary Material

Impact of *pe_pgrs33* Gene Polymorphisms on *Mycobacterium tuberculosis* Infection and Pathogenesis

Serena Camassa[1], Ivana Palucci[1], Raffaella Iantomasi[2], Tiziana Cubeddu[3], Mariachiara Minerva[1], Flavio De Maio[1], Samuel Jouny[2], Elisa Petruccioli[4], Delia Goletti[4], Francesco Ria[5], Michela Sali[1], Maurizio Sanguinetti[1], Riccardo Manganelli[6], Stefano Rocca[3], Priscille Brodin[2] and Giovanni Delogu[1*]

*** Correspondence:** Giovanni Delogu, giovanni.delogu@unicatt.it

1. Supplementary Tables

Supplementary Table 1. Details of the primers used in this study.

Supplementary Table 2. Details of the 19 *pe_pgrs33* alleles identified in this study.

2. Supplementary Figures

Supplementary Figure 1. Complementation of *Mtb*Δ33::33^{all3} assessed by PCR amplification. Complementation of the *Mtb*Δ33 mutant with the 33^{all3} allele under the control of its native promoter was verified by performing two different PCRs (lines 2 and 6). Two positive controls were used in all PCRs, the genomic DNA of *Mtb* H37Rv (lines 1 and 5) and the recombinant pMV306 plasmid specifically used for the *Mtb*Δ33 mutant strain transformation (lines 3 and 7). Negative controls (water) are reported in lines 4 and 8. PCR1 was performed by using PG335Hn-338bp and 18c3AXb primers, while PCR2 was performed by using PMV306 MF and PMV306 MR primers **(Supplementary Table 1)**. All genetic variations, including the 1bp deletion in position 1014bp previously ascribed in this study to the 33^{all1}, 33^{all2} and 33^{all3} alleles, were further verified by sequencing 33^{all3} in *Mtb*Δ33::33^{all3} (Wang et al. 2011; Talarico et al. 2005) (data not shown).

Supplementary Figure 2. Superlineage-based distribution of 135 MTBC clinical strains isolated in Rome. Each superlineage is specifically colour-coded, as previously described (Comas and Gagneux 2011). Percentage of the MTBC lineages belonging to the superlineage 4 is also showed and colour-coded by different shades of red and includes clinical isolates designated by “?”, likely belonging to the T-specific lineage of the Euro-American superlineage (Allix-Beguec et al. 2008).

Supplementary Figure 3. Antigenicity plot of PE_PGRS33 encoded by the 33^{all11} and 33^{all3} alleles. Each amino acid sequence was analysed *in silico* by using CLC Main Workbench software (Welling scale, window size 11). Upper in the figure, the three main domains of PE_PGRS33 are indicated (PE, TM or transmembrane and PGRS domains). In orange, the protein encoded by 33^{all11} (498aa); in green, the protein encoded by 33^{all3} (374aa). Green dashed lines delimit the PE_PGRS33 region interested by a frameshift deletion occurring in 33^{all3} and shared by 33^{all1} and 33^{all2}.

Supplementary Figure 4. Genetic relationships among the 19 *pe_pgrs33* alleles identified in this study. (A) An unrooted tree based on the genetic variations identified among the 19 *pe_pgrs33* alleles was obtained with the Maximum Likelihood method. For each *pe_pgrs33* allele both the spoligotyping-defined lineage and the number of the respective MTBC clinical isolates are specified. (**B)** Apart from allele 11, which correspond to the *pe_pgrs33* gene of *Mtb* H37Rv, genetic variations of all other *pe_pgrs33* alleles identified in this study are indicated and differently colour-coded, based on the MTBC superlineage or superlineages to which they belong to (Comas and Gagneux 2011).

Supplementary Figure 5. Qualitative histopathological analysis of murine lung sections at day 28 post-infection. Lung sections of mice infected with the *Mtb* H37Rv **(A)**, *Mtb*Δ33::33^{all3} **(B)** and *Mtb*Δ33 strains **(C)** were analysed by using ZN staining. Representative images are reported and show details of pulmonary lesions.

Supplementary Figure 6. Qualitative histopathological analysis of murine lung sections at day 49 post-infection. Lung sections of mice infected with the *Mtb* H37Rv (**A** and **B**), *Mtb*Δ33::33^{all11} **(C** and **D**), *Mtb*Δ33::33^{all3} **(E** and **F)**, *Mtb*Δ33::33^{all6} **(G** and **H)** and *Mtb*Δ33 strains **(I** and **L)** were analysed by using ZN staining. Representative images are reported and show details of pulmonary lesions.

frontiers
