# Supplementary figures and images for: Impact of pe_pgrs33 Gene Polymorphisms on Mycobacterium tuberculosis Infection and Pathogenesis
Source: Front Cell Infect Microbiol. 2017 Apr 21;7:137. doi: 10.3389/fcimb.2017.00137 (PMC5399086; doi:10.3389/fcimb.2017.00137)

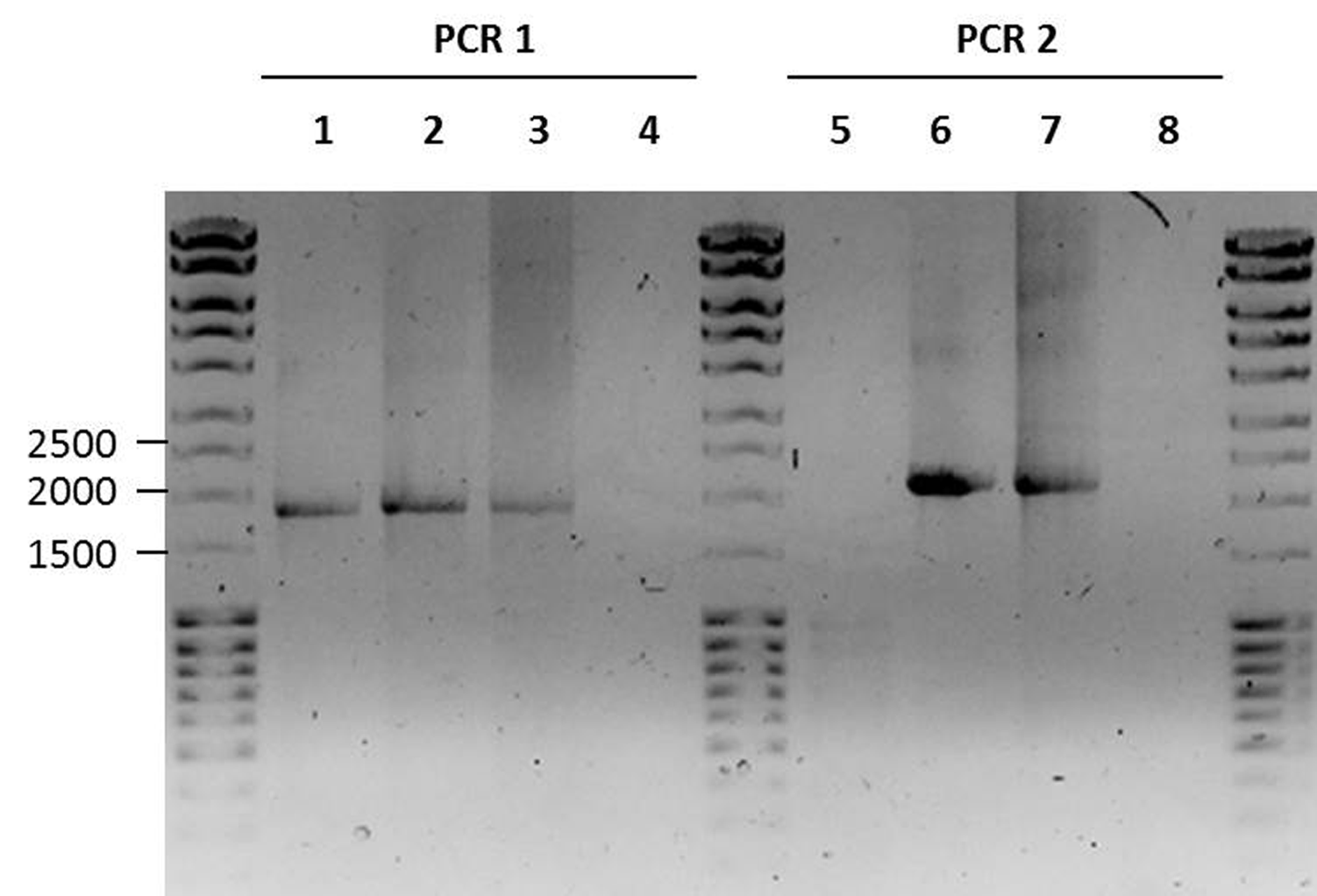

Supplement: Supplementary file 4 [file Image1.TIF]

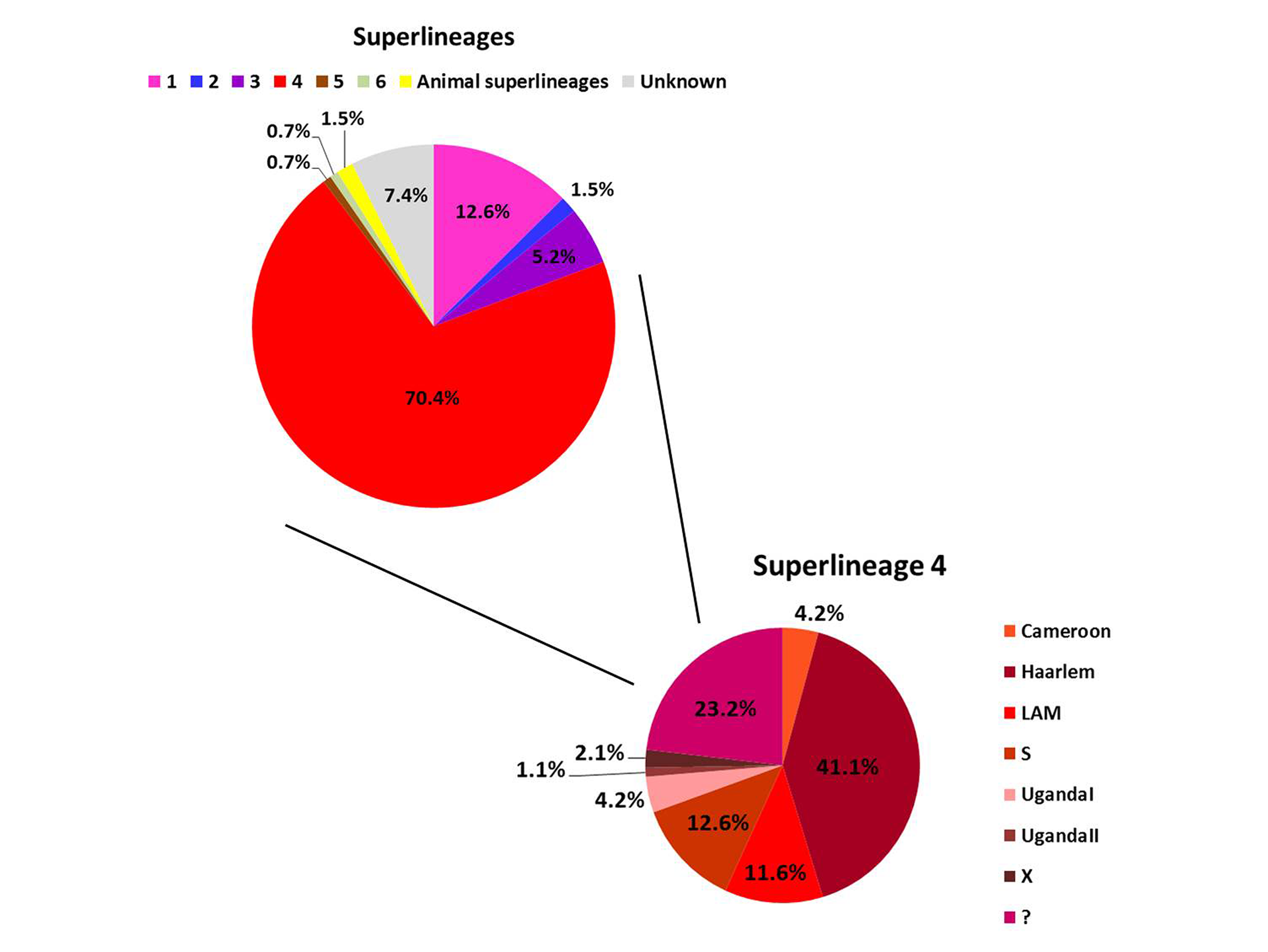

Supplement: Supplementary file 5 [file Image2.TIF]

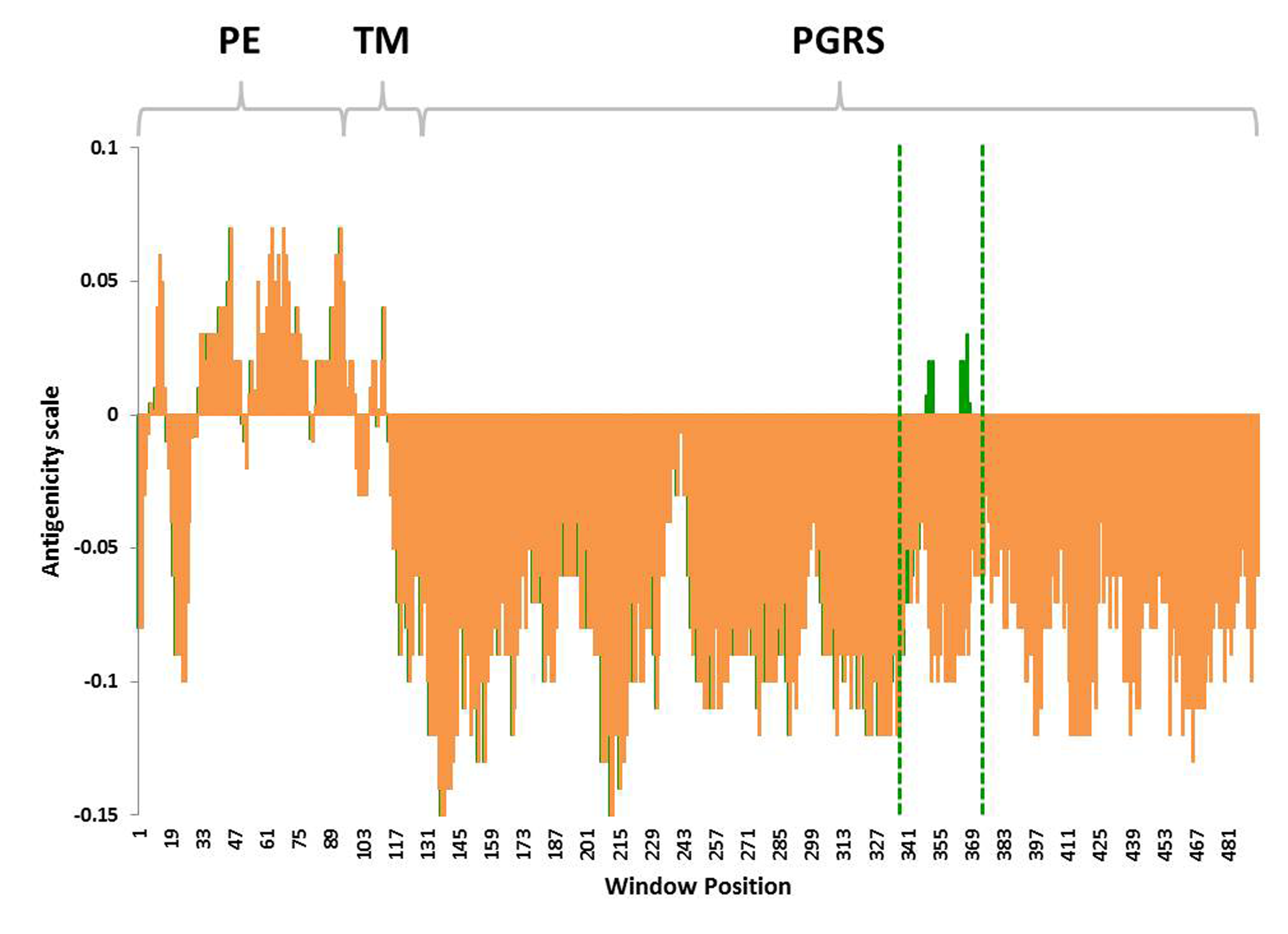

Supplement: Supplementary file 6 [file Image3.TIF]

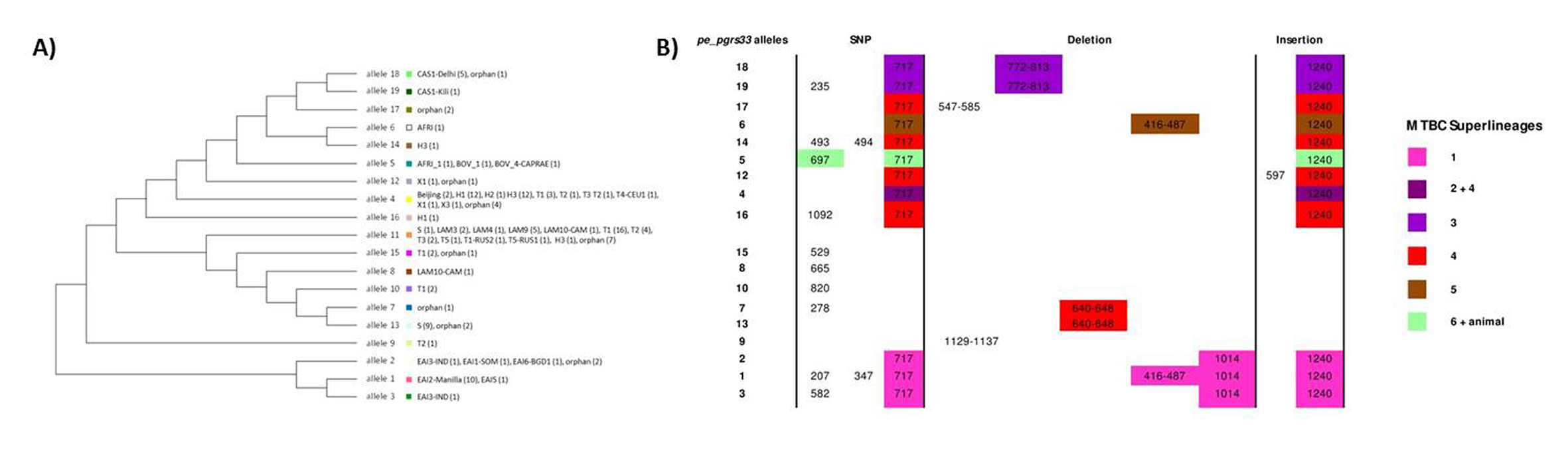

Supplement: Supplementary file 7 [file Image4.TIF]

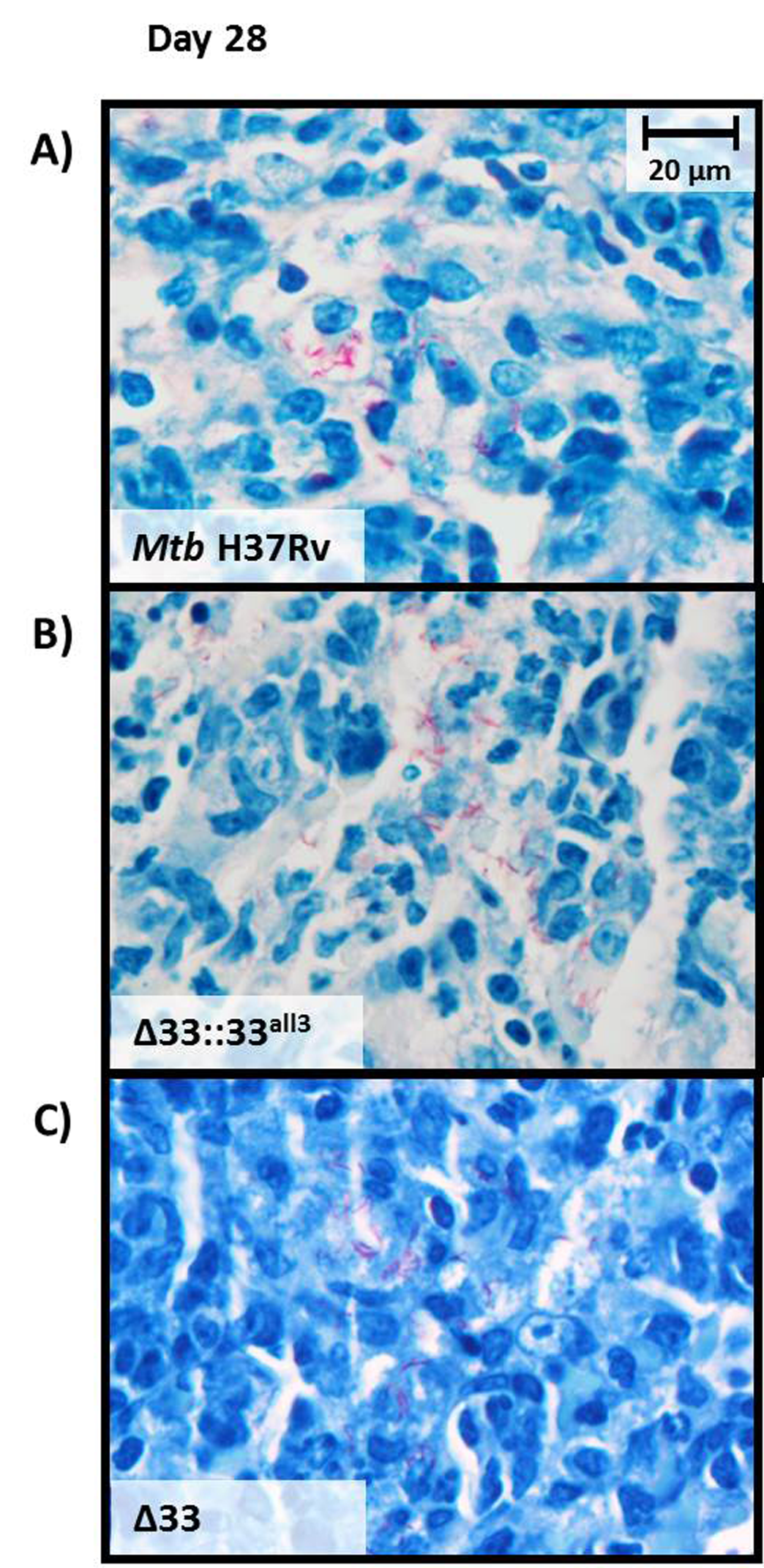

Supplement: Supplementary file 8 [file Image5.TIF]

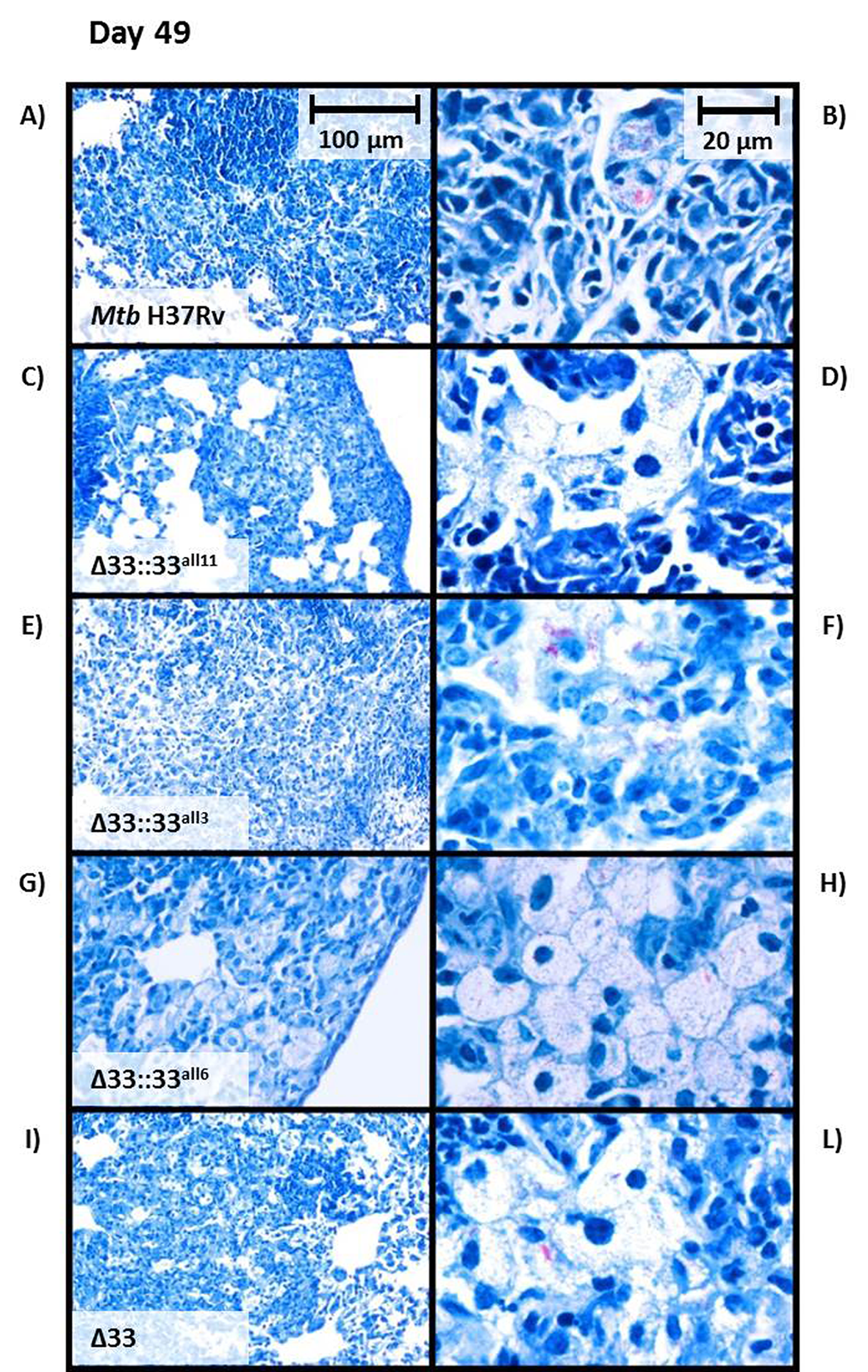

Supplement: Supplementary file 9 [file Image6.TIF]
